# Protocol of the RADIO-STAR trial: a phase 1 safety and dose finding study of hypofractionated radiotherapy to the stellate ganglia for the treatment of ventricular arrhythmia

**DOI:** 10.1136/bmjopen-2025-110958

**Published:** 2026-02-25

**Authors:** Benjamin Mothibe Bussmann, Ben George, Maxwell Robinson, James Grist, Prabakar Sukumar, Ebison Chinherende, Fintan Sheerin, Veni Enzhil, Oliver Rider, Bleddyn Jones, Ami Sabharwal, Neil Herring

**Affiliations:** 1 Department of Physiology, Anatomy and Genetics, University of Oxford, Oxford, UK; 2 Department of Cardiology, Oxford Heart Centre, Oxford University Hospitals NHS Foundation Trust, Oxford, England, UK; 3 GenesisCare UK, Oxford, UK; 4 Department of Radiotherapy Physics, Oxford University Hospitals NHS Foundation Trust, Oxford, England, UK; 5 Oxford Centre for Clinical Magnetic Resonance Research, University of Oxford, Oxford, England, UK; 6 Department of Radiology, Oxford University Hospitals NHS Foundation Trust, Oxford, England, UK; 7 Department of Neuroradiology, Oxford University Hospitals NHS Foundation Trust, Oxford, England, UK; 8 Department of Oncology, Oxford University Hospitals NHS Foundation Trust, Oxford, England, UK

**Keywords:** Cardiomyopathy, Pacing & electrophysiology, Adult radiotherapy

## Abstract

**Introduction:**

Sympathetic activation is the hallmark of cardiac disease, driving disease progression and triggering ventricular arrhythmia (VA). Despite optimal medical therapy, many patients experience recurrent VAs refractory to medical therapy, leading to repetitive implantable cardioverter defibrillator (ICD) therapy, worse quality of life and adverse outcomes. Cardiac sympathetic denervation (CSD) through surgical removal of the stellate ganglia is an effective treatment for refractory VAs but carries a high complication rate. We hypothesise that high precision image guided radiotherapy can be used to target the stellate ganglia to achieve CSD non-invasively.

**Methods and analysis:**

RADIO-STAR (hypofractionated radiotherapy to the stellate ganglia for ventricular arrhythmia) is a first-in-human, phase 1 safety and dose finding study of radiotherapy to the stellate ganglia in patients with recurrent VAs. Patients with structural heart disease requiring recurrent ICD therapy for VAs are invited to undergo radiotherapy bilaterally to their stellate ganglia with a predetermined sample size of n=13. Radiotherapy dose will be determined by a prespecified dose escalation protocol. The primary outcome is safety defined as any treatment-related grade 3–5 toxicity occurring within 6 months of radiotherapy treatment, as defined by the Common Terminology Criteria for Adverse Events or any treatment-related side effects detected on patient symptom questionnaires and clinical examination during study visits. Secondary outcome measures to evaluate feasibility and efficacy include ability to safely deliver radiotherapy and consequent changes in circulating catecholamines and neuropeptide-Y, heart rate variability, structural changes in the stellate ganglia on MRI imaging and ICD therapy burden.

**Ethics and dissemination:**

This study has received ethical approval by the South Central—Oxford B Research Ethics Committee (REC/SC/0005). Study findings will be submitted for publication in peer-reviewed scientific journals and presented at national and/or international scientific conferences.

**Trial registration number:**

ISRCTN49861434.

**STRENGTHS AND LIMITATIONS OF THIS STUDY**

- This first-in-human study will establish the safety and feasibility of radiotherapy to the stellate ganglia and provide key data on radiotherapy dose selection for future randomised controlled trials evaluating the efficacy and durability of radiotherapy-induced cardiac sympathetic denervation for the prevention of ventricular arrhythmia.
- As an early-phase, safety-focused investigation, this study is not powered to assess clinical efficacy, so the evaluation of treatment effects on arrhythmia burden is beyond the scope of this study.
- In the absence of a control group, changes in implantable cardioverter defibrillator (ICD) therapy burden following radiotherapy may be confounded by concurrent clinical interventions, including ventricular tachycardia ablation, adjustments to antiarrhythmic medications or reprogramming of ICD settings, and should be interpreted with caution.
- The maximum follow-up period of 6 months limits assessment of long-term outcomes but is sufficient for initial safety assessment and radiotherapy dose evaluation.
- Observed changes in heart rate variability, circulating biomarkers or stellate ganglion appearance on MRI represent indirect evidence of reduced cardiac sympathetic tone and should be interpreted as hypothesis-generating only.

## Introduction

 Ventricular arrhythmias (VA) are life-threatening events and the most common cause of sudden cardiac death (SCD).^1^ SCD accounts for up to 20% of deaths in the western world.^2^ Patients with structural heart disease such as heart failure with reduced ejection fraction (HFrEF) are at particularly increased risk of SCD. There are approximately 6.7 million Americans living with heart failure, and 1 in 4 are expected to develop heart failure within their lifetime.^3^ While advances in medical therapy have improved outcomes in HFrEF, the incidence of SCD remains stubbornly high.^4^

In patients at very high risk of SCD, the implantable cardioverter defibrillator (ICD) improves mortality.^5 6^ While ICDs are effective at treating VAs as they occur, and thus reducing mortality associated with SCD, they cannot prevent VAs from occurring in the first place. Patients with incessant VAs thus experience recurrent ICD therapies associated with increased mortality^7^ and significant impact on quality of life.^8 9^ Prevention of VAs is thus vital.

β-blockers are the only class of antiarrhythmic medication that reduce mortality due to VAs in heart failure.^10 11^ While other classes of antiarrhythmic drugs can reduce VAs, they lack a mortality benefit,^5 12 13^ are associated with side effects^14 15^ and can even lead to excess mortality through proarrhythmic effects.^16^,^18^ Catheter ablation can be effective at reducing VAs,^19^ but up to a third of patients have recurrence of VAs, and clinical trials of VT ablation have to-date failed to demonstrate a mortality benefit.^20^,^24^ Thus, despite all contemporary treatments, the incidence of arrhythmia and death in patients with HFrEF remains high, at around 5–10% per year.^5 6 25 26^ For such patients few remaining options exist. Heart transplantation may also be considered but is limited by organ availability and stringent candidate selection criteria.^27^

The heart is densely innervated by a network of autonomic neurons^28 29^ which control every aspect of cardiac function including electrophysiological properties of cardiac tissue.^30^,^34^ Conceptually, the autonomic nervous system can be considered to exert its influence through two opposing arms; the sympathetic system which increases inotropy, chronotropy, lusiotropy and dromotropy, and the parasympathetic system which decreases these parameters.^35 36^ In health, this system exists in a state of intricate balance to maintain cardiac homeostasis.^37^ However, the hallmark of cardiac failure, irrespective of aetiology, is a loss of this balance resulting in sympathetic activation and parasympathetic withdrawal.^38 39^ While initially, sympathetic activation preserves cardiac output in response to acute cardiac insult, over time it becomes maladaptive and contributes to disease progression.^40 41^

Sympathetic tone is highly proarrhythmic. β-adrenergic stimulation alters cardiomyocyte repolarisation through protein kinase A and calcium/calmodulin-dependent protein kinase II mediated phosphorylation of calcium handling proteins and ion channels. Activation of L-type calcium channels, the ryanodine receptor 2, sodium/calcium exchange and smooth endoplasmic reticulum calcium ATPase leads to calcium loading in cardiomyocytes.^42^ Calcium loading predisposes to delayed afterdepolarisations which are triggers for many pathological arrhythmias.^32 43 44^ Furthermore, by increasing the slow outward potassium current, β-adrenergic stimulation shortens action potential duration (APD),^45^ which decreases refractory period and helps sustain re-entry circuits that underlie most VAs.

Recently, the importance of sympathetic cotransmitters such as neuropeptide-Y (NPY) has also been established. Notably, NPY has strong proarrhythmic effects even in the presence of maximal β-blockade^46 47^ mediated by ventricular myocyte Y1 receptor promotion of calcium signalling. In fact, NPY may serve as a potential biomarker of arrhythmic risk and has been shown to predict arrhythmia and death in chronic heart failure and following myocardial infarction.^46 48 49^

The majority of cardiac sympathetic innervation arises from the stellate ganglia; a fusion of the lower cervical and first thoracic sympathetic ganglia.^50 51^ In vivo, stimulation of the stellate ganglia decreases fibrillatory threshold and induces VA.^3452^,^54^ Additionally, regional differences in cardiac sympathetic innervation produce regional heterogeneity in cardiac myocyte electrical properties which predisposes to arrhythmia.^55^,^58^ Indeed, stellate ganglion stimulation increases the time interval from the peak to the end of the electrocardiographic T wave, a marker of cardiac dispersion of repolarisation and an independent predictor for risk of SCD.^33^ In ischaemic heart disease, these effects are further compounded by sympathetic nerve sprouting at infarct border zones, super sensitivity in infarcted myocardium^59^ and differing responses to sympathetic stimulation in ischaemic versus normal myocardium, enhancing regional differences in ventricular refractories and predisposing to arrhythmia.^60^ Heterogeneity in APD and activation-recovery intervals is further amplified by sympathetic stimulation of the sinoatrial node, which through increases in heart rate promotes discordant calcium-driven APD alternans.^61^

Finally, sympathetic activation inhibits parasympathetic tone through sympathovagal crosstalk, further entrenching autonomic imbalance and limiting the cardioprotective and antiarrhythmic effects of parasympathetic activation.^62^,^68^

Unsurprisingly, methods to reduce sympathetic tone have emerged as promising treatments for VAs. Deep sedation is known to reduce overall sympathetic tone and is effective during arrhythmic storm.^69^ Similarly, injection of local anaesthetic into the epidural space at level T1–T4 where cardiac sympathetic preganglionic fibres emerge (called thoracic epidural anaesthesia) or direct stellate ganglion block can be used to manage arrhythmic storm.^70^,^73^ While these techniques are effective holding measures, a more permanent form of cardiac sympathetic denervation (CSD) can be achieved through surgical removal of the lower half of the stellate ganglia along with T2–T4 sympathetic ganglia.^74^,^76^

In the 1990s, Schwartz and colleagues pioneered surgical CSD as a treatment for patients at high arrhythmic risk due to long QT syndrome (LQTS) and catecholaminergic polymorphic ventricular tachycardias (CPVTs). They showed that patients with recurrent cardiac arrest despite maximal β-blocker therapy could be successfully treated with CSD.^77^,^79^ This led to widespread adoption of surgical CSD^80^,^83^ and its inclusion in clinical guidelines for managing patients with LQTS and CPVT.^84^ Schwartz *et al*^85^ also demonstrated the utility of CSD in ischaemic heart disease. In a small randomised trial, 144 patients with MI complicated by VAs were randomised to placebo, CSD or β-blockers. Over a 22-month follow-up the rate of SCD (21%) in the control group was markedly reduced by both β-blockade and SCD (2.7% and 3.6%, respectively).

However, the use of CSD in HFrEF initially remained limited due to concerns regarding haemodynamic decompensation resulting from removal of sympathetic drive. However, Shivkumar^19^ and colleagues have since dispelled these concerns. They showed that CSD is safe in the presence of HFrEF and that bilateral surgical CSD is more effective at preventing VAs than left-only CSD.^58 74 86^ Indeed, bilateral CSD is strikingly effective, leading to over 60% reduction in VAs in patients refractory to all available treatments, including maximally tolerated doses of β-blockade.^87^ Unfortunately, despite progressive refinements of surgical SCD, including minimally invasive video-assisted thoracoscopic approaches,^83^ the procedure continues to carry high complication rates, limiting its mainstream use.^87 88^ Furthermore, surgery requires long hospital stays often including postoperative intensive care admissions.^89^

Modern image-guided radiotherapy can deliver highly targeted treatments with submillimetre precision and steep dose gradients, allowing for rapid dose fall-off outside the treatment area. This has revolutionised cancer treatment by enabling the safe delivery of high-dose radiotherapy to small tumours while still minimising radiation dose to surrounding healthy tissues.^90^,^92^ We hypothesise that this technology could be used to target the stellate ganglia to achieve CSD non-invasively, resulting in fewer complications while maintaining efficacy.

## Methods and analysis

This protocol is reported in accordance with the Standard Protocol Item Recommendations for Interventional Trials 2025 guidance^93^ (online supplemental file 2).

### Study design

**Figure 1:**
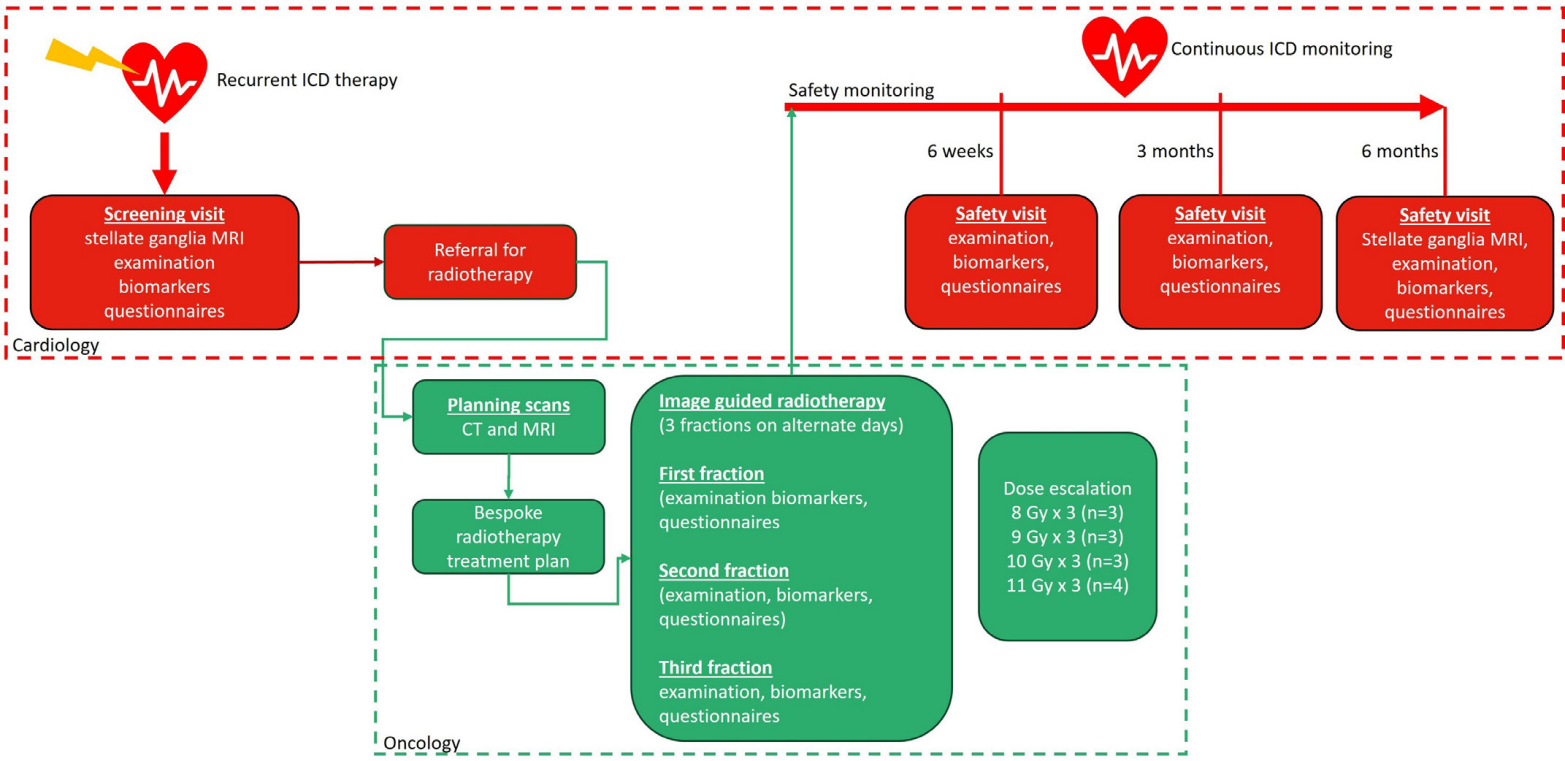
Flow diagram illustrating overall study design of the RADIO-STAR trial. ICD, implantable cardioverter defibrillator; RADIO-STAR, hypofractionated radiotherapy to the stellate ganglia for ventricular arrhythmia.

RADIO-STAR (hypofractionated radiotherapy to the stellate ganglia for ventricular arrhythmia) is a phase 1 clinical trial to evaluate the safety, feasibility and optimal dose of image guided radiotherapy to the stellate ganglia to achieve CSD. The study comprises a multidisciplinary team including oncologists, neuroradiologists and cardiologists. The overall study design is illustrated in figure 1, and the full trial protocol is available as an online supplement (Data online supplemental file 2)

**Figure 2:**
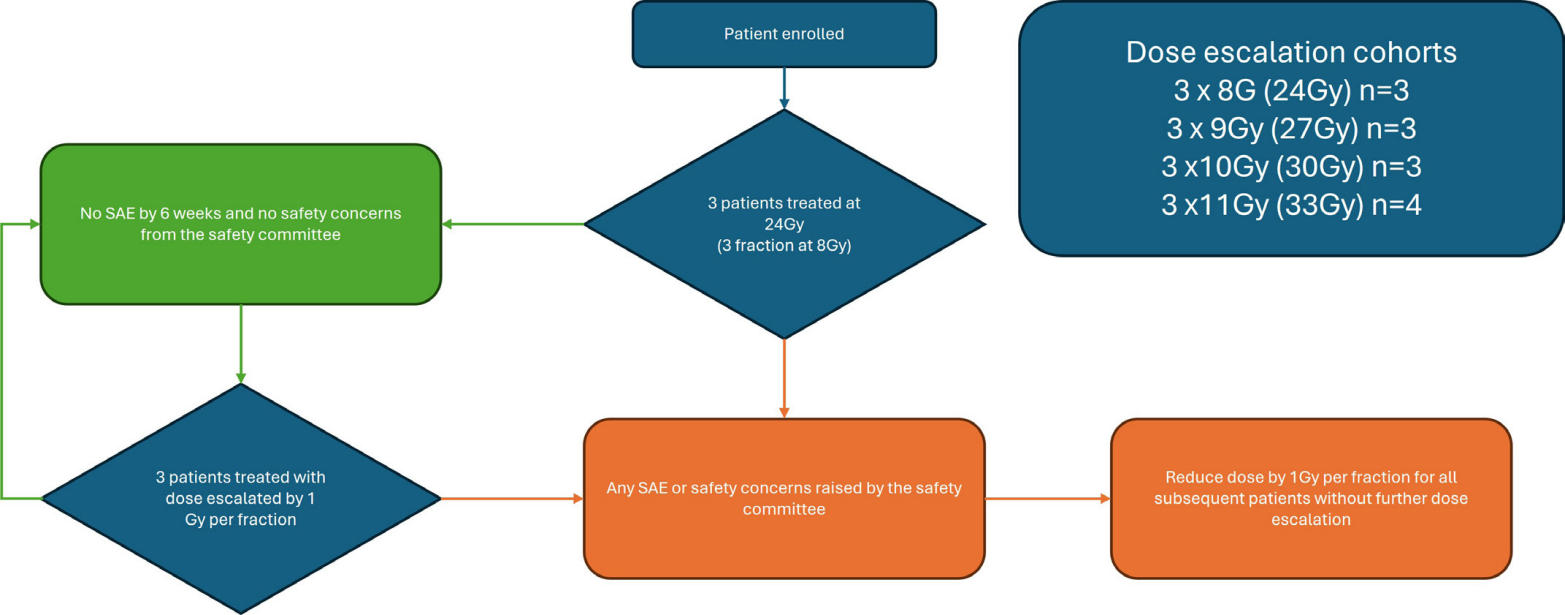
Dose escalation protocol. The first three participants will be treated at a total dose of 24 Gy as 8 Gy per fraction for three fractions on alternate days. If no related SAEs occur in this cohort, the dose will be escalated by 1 Gy per fraction for the subsequent three patients. This will be repeated up to a maximum dose of 11 Gy per fractions (total 33 Gy) for the final four patients. There will be a minimum of 6 weeks between the last participant treated at each dose before escalating the next dose to allow for detection of adverse events at participants’ 6 week follow-up visit. Before each dose escalation, an independent safety committee will review all patient data and radiotherapy doses will only be escalated if there are no safety concerns. In the event of a related SAE, no further fractions would be given to that patient, and the dose would be reduced by 1 Gy per fraction for all subsequent patients (up to a total of 13 patients) without further dose escalation. An early stopping rule will be set to halt enrolment if four out of the first eight patients develop a related SAE. SAEs in this trial are defined as any Grade 5, Grade 4 or Grade 3 toxicity requiring hospitalisation as defined by the Common Terminology Criteria for Adverse Events (V.5.0). SAE, serious adverse event.

All participants will receive radiotherapy bilaterally to the lower half of their stellate and T1–2 sympathetic ganglia. Evidence suggests that the risk of Horner’s syndrome and hyperalgesia may be mitigated by sparing the cranial portion of the stellate ganglion and the T3–T4 paravertebral ganglia while still achieving sufficient CSD.^51 74 76^ Radiotherapy will be delivered in three fractions from a total starting dose of 24 Gy (the maximum upper limit for radiation dose to neuronal structures such as the brachial plexus),^94^ to a maximum dose of 33 Gy. Radiotherapy doses are derived from historical literature targeting other neuronal structures such as the trigeminal ganglia.^95^,^100^ The ‘neurosurgical approach’ involving very large single-fraction doses of up to 90 Gy, as commonly used to treat trigeminal neuralgia,^101^ is likely to be higher than required. Rather, lower subablative doses to achieve functional ganglion modification instead of its destruction may be adequate.^102^ Furthermore, this would reduce the risk of off-site neurological sequelae and minimise radiation dose to nearby organs at risk (OAR) and ICD devices. Radiotherapy will be delivered under either CT (Varian TrueBeam) or MRI (MRIdian MR-Linac) image guidance. Treatment dose will be prespecified by an ‘up-and-down’ dose escalation protocol^103 104^ designed to identify the maximum tolerated radiotherapy dose (figure 2).

### Objectives

The primary objective of this study is to determine if image-guided radiotherapy to the stellate ganglia is feasible and safe.

### Patient population

This study aims to recruit thirteen patients with structural heart disease who experience recurrent VAs requiring ICD therapy despite optimised medical treatment. Patients receiving care at the John Radcliffe Hospital, Oxford, UK, who meet the eligibility criteria (see box 1) will be considered.

Box 1 **Study inclusion and exclusion criteria**

**Inclusion criteria**

- Participant is willing and able to give informed consent for participation in the study.
- Male or female, aged at least 18 years old.
- Known diagnosis of structural heart disease defined as any patient with impaired left ventricular ejection fraction (less than 55%) due to any cause including ischaemic cardiomyopathy, dilated cardiomyopathy, hypertrophic cardiomyopathy or arrhythmogenic cardiomyopathy.
- MRI-compatible ICD device implanted a minimum of 6 months ago, under follow-up at Oxford University Hospitals NHS Foundation Trust.
- Experienced more than one appropriate ICD therapy (shocks or ATP) for ventricular arrhythmia in the last 6 months.
- Established on optimal guideline-based medical therapy for heart failure and ventricular arrhythmia.

**Exclusion criteria**

- Device radiation dose or nearby organs at risk exceeding the limit for low-risk radiotherapy as defined by the UK Consensus and AAPM 2019 guidelines.
- Severe kidney impairment (eGFR less than 30) which would prevent the safe use of iodine contrast during CT scans.
- Female patients who are pregnant, lactating or planning pregnancy during the study period.
- Patients who are terminally ill, inappropriate for intervention or unable to consent.
- Any impediment to communication which, in the opinion of the investigator, might prevent the investigator communicating effectively with the patient during the study, which could cause a safety or reliability concern.
- Any other condition which, in the opinion of the investigator, might affect the safety of the participant or reduce the reliability of the study result.
- Involvement in any other research project where the procedures would affect the outcomes of this study.
- Any condition preventing the safe use of MRI imaging (metal clips or metallic foreign body, prior injury to the eye involving fragments of metal, prior shrapnel injuries, any other metallic or electronic implants affected by the magnetic field, history of severe claustrophobia).

AAPM, American Association of Physicists in Medicine; ATP, Anti-Tachycardia Pacing; eGFR, estimated glomerular filtration rate; ICD, implantable cardioverter defibrillator.

### Study plan

Participation in this study will consist of nine visits, including an initial screening visit, followed by eight study visits over the course of approximately 6 months (figure 1). A schedule of events of all study procedures is available as part of the full trial protocol on-line (data online supplemental file 2).

### Visit 1: screening visit and screening 1.5T MRI

Participants will be asked to provide written informed consent prior to screening. Patient age, height, weight, gender, prior diagnosed comorbidities, cardiac function, smoking status, Canadian Cardiovascular Society angina grade, New York Heart Association heart failure severity and medication history will be recorded. The Kansas City Cardiomyopathy Questionnaire (KCCQ-23) will assess baseline quality of life scores, and routine cardiovascular and neurological examination will establish baseline clinical status. A 12-lead ECG, 10 min heart rate variability (HRV) measurements, postural blood pressures and baseline blood tests will also be taken.

**Figure 3:**
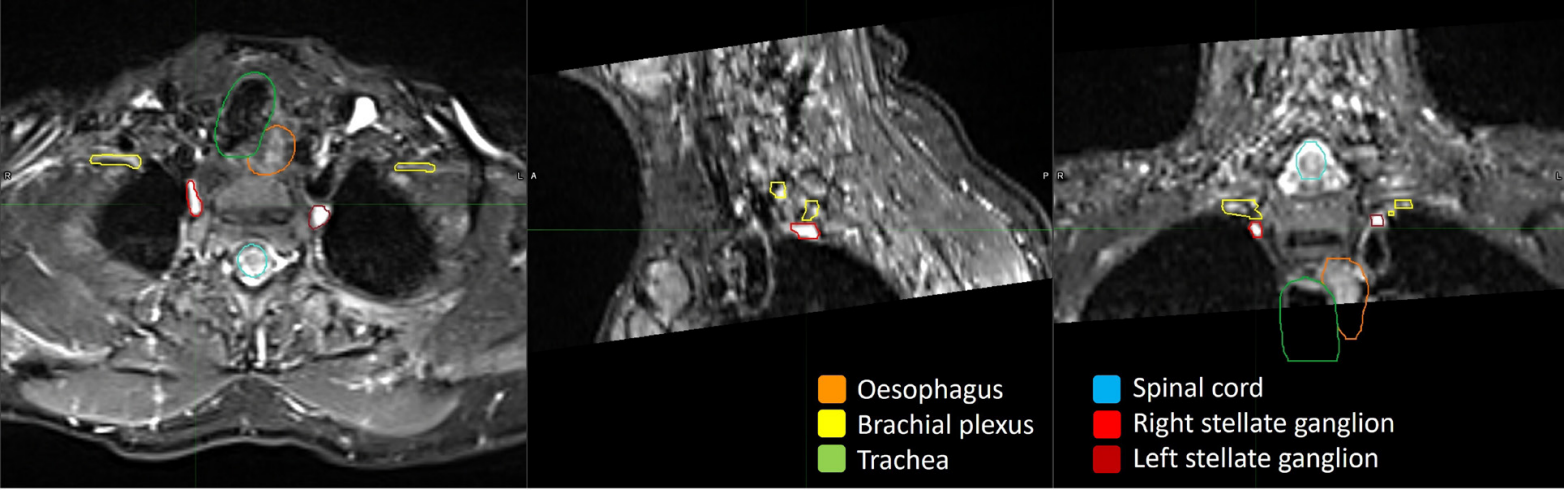
1.5T MRI diagnostic imaging of the stellate ganglia. A T2 weighted turbo spin echo MRI with short tau inverted recovery fat suppression of a participant with a left sided ICD device. Planning target volumes are contoured over the stellate ganglia and key dose-limiting organs. ICD, implantable cardioverter defibrillator.

An MRI scan of the stellate ganglia will be performed to ensure adequate visualisation of the stellate ganglia. We have undertaken extensive pilot work to optimise T2-weighted turbo spin echo MRI sequences with short tau inversion recovery fat suppression to clearly visualise the stellate ganglia while minimising device-related artefact (figure 3). This imaging will be used for preliminary planning to evaluate whether the intended treatment dose can be delivered to the stellate ganglion without exceeding the maximum tolerated doses for the ICD device or adjacent OAR. If it is not possible to deliver radiotherapy without exceeding the limit for low-risk radiotherapy to OAR—as defined by the UK and American Association of Physicists in Medicine 2019 consensus^94105^,^107^—the patient will be withdrawn.

### Visit 2: radiotherapy team visit and study consent

Only patients with adequate stellate ganglia visualisation and acceptable radiotherapy treatment plans will proceed with the study. This process will begin with an introductory visit to the radiotherapy team. Patients will receive detailed counselling about the radiotherapy procedure, potential side-effects and will be asked to provide written informed consent for full study enrolment.

### Visit 3: planning scans, radiotherapy delineation and treatment planning

A planning contrast CT scan of the stellate ganglia necessary for further treatment planning will be performed. Participants receiving MR-guided radiotherapy will also undergo an additional 0.35T MRI planning scan performed on the MRIdian MR-Linac. Both images will be fused with the pretreatment diagnostic MRI to aid organ contouring. Accurate positioning for the planning scan and subsequent treatment will be facilitated by a 5-point thermoplastic mask immobilising patient head and shoulders. Optimal fields of view for CT and MRI scans (if required) will be from the patient’s inferior orbital ridges to carina. Imaging slice thickness will be 2 mm on CT and 1.5 mm on 0.35T MRI.

**Figure 4:**
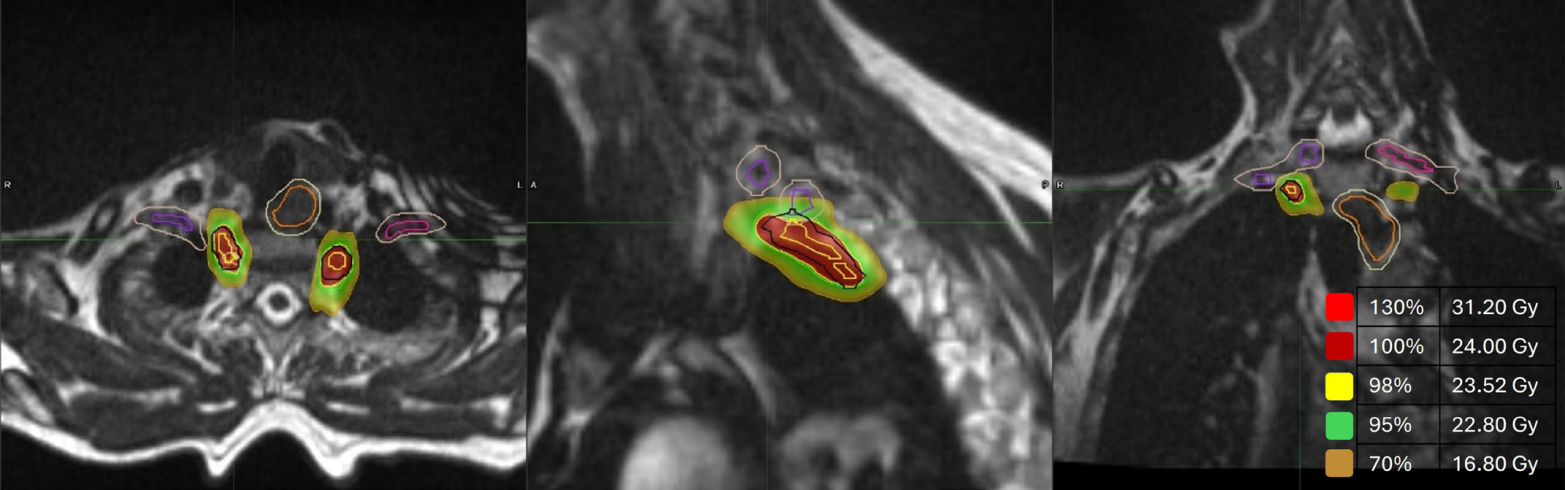
Radiotherapy doses displayed on the 0.35T Trufi planning image. Radiotherapy isodose lines over the stellate ganglia for a patient with target total dose of 24 Gy. Also displayed are the contours over the oesophagus (orange) and brachial plexus (purple) with a surrounding planning organ at risk volume.

The stellate, T1 and T2 ganglia will be identified with the aid of the baseline 1.5T MR images coregistered with the CT and 0.35T MR planning images. The clinical target volumes (CTVs) are defined as the lower half of the stellate ganglion, T1 ganglion and T2 ganglion bilaterally (CTV-right and CTV-left). A planning target volume (PTV) will be created around each CTV with an additional 3 mm margin to account for any uncertainties in treatment planning and delivery (PTV-right, PTV-left). The prescription dose will be prescribed to this volume, such that 95% of the volume should receive 100% of the dose (see figure 4).

Dose distribution will be heterogeneous, with a moderately high maximum dose (up to 120%) at the centre of the PTV. Dose gradients will be steep outside of the PTVs to minimise dose to adjacent OAR. PTV coverage will be compromised to ensure adjacent OAR dose constraints are achieved (as shown in table 1).^94 106 107^ The minimum acceptable volume (V100%) coverage of PTV-right and PTV-left individually is 75%.

**Table 1:** Dose constraints to organs at risk

Organ at risk	Metric	Total dose over three fractions
Brachial plexus	D(0.1 cc)	≤24.0 Gy
Chest wall	D(0.1 cc)	≤36.9 Gy
	D(30.0 cc)	≤30.0 Gy
Great vessels	D(0.1 cc)	≤45.0 Gy
Oesophagus	D(0.1 cc)	≤25.2 Gy
Lungs	V(20 Gy)	≤15%
Spinal cord	D(0.1 cc)	≤20.3 Gy
Skin	D(0.1 cc)	≤33.0 Gy
	D(10.0 cc)	≤30.0 Gy
Trachea	D(0.1 cc)	≤30.0 Gy
Carotid artery	D(0.03 cc)	≤32.5 Gy
Thyroid lamina	D(0.03 cc)	≤30.0 Gy
ICD generator	D (0.03 cc)	≤5.0 Gy

D(xx cc) <yy Gy denotes the maximum dose (yy Gy) that can be given to a specified volume of the organ (xx cc), with the remaining volume receiving less than the threshold dose. V(yy Gy) <xx% denotes the maximum critical percentage volume of the organ (xx %) that can receive the specified threshold dose (yy Gy).

ICD, implantable cardioverter defibrillator.

### Visits 4–6: hypofractionated radiotherapy to the stellate ganglia

Radiotherapy will be delivered in three fractions on alternate days, over the course of 1 week. Before and after each fraction of radiotherapy, cardiovascular and neurological examinations, ECGs, HRV measurements, postural blood pressures and symptoms questionnaire scores will be recorded. A blood sample will be taken after each fraction. This gives the opportunity to pause radiotherapy delivery should side effects develop. Treatment will be delivered with daily image guidance, via bony anatomy adjacent to the PTV, adjustment of the couch position and matching of the treatment target to within the PTV. The position of the brachial plexus will also be monitored to confirm it remains within the OAR dose constraint.

### Visits 7–9: safety visits

Study follow-up will comprise three study visits at 6 weeks, 3 months and 6 months following completion of radiotherapy. During each study visit, cardiovascular and neurological examinations, ECGs, HRV measurements, postural blood pressures and symptoms questionnaire scores will be recorded and a blood sample taken. At the final 6-month study visit, participants will also complete the KCCQ-23 and undergo a final stellate ganglion 1.5T MRI. As part of study follow-up, a record will be kept of patients’ arrhythmia management by their usual clinical team including cardiac ablation procedures, changes in cardiac medications and alterations to ICD programming parameters.

### ICD interrogation and monitoring

Throughout the study duration, all patients ICDs will be under remote monitoring as part of routine clinical care. In addition, ICDs will be interrogated at each study visit to screen for device-related complications resulting from radiotherapy.

### Outcome measures

The primary outcome of this study is safety, and this study is powered to detect serious adverse events (SAE) as our primary endpoint. SAE is defined as any related and unexpected grade 3 toxicity requiring hospitalisation, grade 4 toxicity or grade 5 toxicity as defined by the Common Terminology Criteria for Adverse Events V.5.0. Relatedness of any SAE to the trial intervention will be decided by an independent safety committee. The presence of less severe treatment-related side effects will be assessed through a dedicated symptom questionnaire (data online supplemental file 3) and full cardiovascular and neurological examination by a trained clinical member of the study team at every radiotherapy and follow-up visit. The symptom questionnaire includes a comprehensive list of symptoms that are commonly reported following surgical CSD^88 108^ and radiotherapy treatment for head and neck cancers.^109^ Any radiotherapy-related side effects will be correlated with total radiation dose and biological effective dose to the relevant OAR.

Secondary outcome measures to assess efficacy include measuring radiotherapy-induced changes in circulating norepinephrine and NPY levels, ICD therapy burden, HRV and structural changes in the stellate ganglia detected on 1.5T MRI imaging (as shown in table 2). Finally, the effect on patient quality of life will be assessed by the KCCQ-23 which has been extensively validated to assess health status and quality of life in patients with a range of cardiomyopathies.^110^,^113^

**Table 2:** Study outcome measures

	Outcome	Measure
Primary outcome		
Safety	Treatment-related SAEs	SAEs are defined as any grade 3 toxicity requiring hospitalisation or any grade 4–5 toxicity in the first 6 months as defined by the Common Terminology Criteria for Adverse Events. The relatedness of any SAE will be determined by an independent safety committee.
	Treatment-related side effects not meeting SAE criteria	Patient reported symptoms on symptom questionnaire (data online supplemental file 3). Clinical findings on full cardiovascular and neurological examination.
Secondary outcomes		
Feasibility	Ability to target the stellate ganglia at the protocol prespecified dose	Acceptable visualisation of stellate ganglia on 1.5T baseline MRI. Ability to deliver protocol-specified radiotherapy dose without exceeding the maximum tolerated doses for the ICD device or adjacent organs at risk
Efficacy	Physical modification of the stellate ganglia following treatment	Qualitative and quantitative changes in stellate ganglion morphology at 6-month follow-up 1.5T MRI compared with baseline
	Changes in HRV following treatment	The following HRV metrics will be calculated from R–R intervals from a 10 min, supine, spontaneously breathing recording at every study visit: Frequency domain: power in the LF (0.04–0.15 Hz) and HF (0.15–0.4 Hz) spectral bands, and LF/HF ratio. Time domain: standard deviation of normal–normal intervals (SDNN), root mean square of successive differences (RMSSD), percentage of successive normal–normal intervals that differ by more than 50 ms (pNN50).
	Changes in biomarkers of sympathetic activity following treatment	Circulating plasma epinephrine, norepinephrine and neuropeptide-Y at 6 weeks, 3 months and 6 months compared with baseline.
	Changes in arrhythmia burden following treatment	Number of ventricular arrhythmias requiring ICD therapy in the 6 months preceding radiotherapy compared with 6 months post radiotherapy.
	Changes in patient-reported quality of life following treatment	KCCQ-23 at 6 months post radiotherapy compared with baseline.

HF, high frequency; HRV, heart rate variability; ICD, implantable cardioverter defibrillator; KCCQ-23, Kansas City Cardiomyopathy Questionnaire; LF, low frequency; SAEs, serious adverse events.

### Sample size calculation

This study is designed to ensure the SAE rate of radiotherapy to the stellate ganglia does not exceed 34%^87^ as its primary outcome. We calculated sample size using the Wald approximation ensuring that the upper bound of the 95% CI (Confidence Interval) for observed adverse events would not exceed 34%. Assuming an observed SAE rate of 16% (by subtracting rates of surgical complications from overall complications rates associated with surgical CSD) we require 13 participants to ensure that the upper limit of the 95% CI around the observed SAE rate would fall below 34%.

### Statistical analysis

For the primary endpoint analysis, the observed SAE rate will be reported with a 95% CI. The decision criterion is whether the upper bound of this CI is below 34%.

Secondary and exploratory outcomes, including biomarkers, HRV, morphological stellate ganglion changes on MRI and KCCQ-23 scores, will be summarised descriptively using appropriate summary statistics. Where appropriate, exploratory paired statistical tests may be used to assess within-patient changes from baseline.

### Safety monitoring

As this is an event-driven study, all adverse events will be reviewed by an independent safety monitoring committee consisting of a consultant cardiologist specialising in cardiac devices and electrophysiology and a consultant clinical oncologist specialising in the radiotherapy treatment of head and neck cancers. As per the dose escalation protocol (figure 1), the committee will routinely meet after the completion of each dose of the dose escalation protocol and dose escalation will only occur once the committee is satisfied with safety. In addition, if any SAE is detected during study follow-up visits, this will trigger a safety committee review and no further participants will receive radiotherapy pending outcome of this review.

### Ethics and dissemination

The study design and research protocol were approved by the South Central—Oxford B Research Ethics Committee (REC 24/SC/005) and informed consent (Data online supplemental file 4) will be obtained from all participants. This study is being conducted in accordance with UK laws, Good Clinical Practice and the Declaration of Helsinki 2002. Findings will be published in peer-reviewed journals and presented at local, national and international meetings and conferences to publicise and explain the research to clinicians, commissioners and service users.

## Discussion

Consistently, measures of autonomic function have revealed a state of sympathetic activation and vagal withdrawal in cardiac disease,^38 39 114^ which is predictive of mortality and SCD in patients with cardiac disease.^115^,^119^ Unsurprisingly, autonomic neuromodulation has become an attractive target in the search for novel therapeutic strategies in cardiac disease, particularly in heart failure.^120 121^

Attempts to harness the cardioprotective and anti-arrhythmic effects of parasympathetic tone through vagal nerve stimulation have proven challenging. The net cardiac effect of stimulating the vagus nerve, which contains both afferent and efferent nerve fibres, reflects a complex interaction between afferent mediated central inhibition of parasympathetic tone and the direct cardiac effects of efferent fibre stimulation.^122^ Randomised clinical trials of vagal nerve stimulation, each using different stimulation protocols, have resulted in discrepant results and failed to demonstrate substantial clinical benefits in humans.^123^,^126^

In contrast, targeting the sympathetic nervous system has proven to be a reliable approach, particularly in preventing VAs. Reducing sympathetic tone through deep sedation or stellate ganglion block is a common guideline endorsed strategy for the management of arrhythmic storm and is associated with consistent reduction in arrhythmia burden.^72 127^ Similarly, a permanent reduction of sympathetic tone through surgical CSD is guideline endorsed for the prevention of SCD in patients with LQTS and CPVT with a similar level of recommendation as ICD implantation.^127^ Increasingly, surgical CSD is also being used to prevent VAs in patients structural heart disease of all aetiologies.^74 87 128^

The stellate ganglia are an appealing therapeutic target, as the majority of cardiac sympathetic innervation originates here, and they undergo extensive remodelling in cardiac disease that favours sympathetic activation. In stellate ganglia removed from patients with recurrent VAs, sympathetic neurons are larger in size, have increased synaptic density, demonstrate signs of oxidative stress and have adrenergic profiles in keeping with sympathetic activation. This is accompanied by proinflammatory changes such as neutrophil and T-cell infiltration, as well as activation of satellite glia.^129 130^ In animal models of cardiovascular disease, sympathetic neurons in the stellate ganglia show altered ion channel expression predisposing to increased excitability. Single cell messenger RNA sequencing in spontaneously hypertensive rats reveals reduced expression of subunit genes associated with the M current,^131^ an inhibitory potassium current which has important influence on neuron resting potential and restricting neuron firing.^132^ Similarly, in rats with ischaemic cardiomyopathy, there is enhanced sympathetic excitability due to increased N-Type calcium currents.^133^

Additionally, the role of satellite glial cells in the stellate ganglia in mediating sympathoexcitation is being increasingly recognised. Each sympathetic neuron is associated with its own satellite glial which envelops the cell body and its synaptic connections, forming a distinct functional unit. This close association with both cell bodies and synapses suggests that glial cells can modulate neuronal activity and synaptic transmission.^134 135^ Sympathetic glia are sensitive to acetylcholine (the primary neurotransmitter at the pre–post ganglionic synapse)^136^ and release factors that augment cholinergic transmission, promote synapse formation, contribute to neuronal survival and modulate neuronal metabolism and neurotransmitter homeostats.^137 138^ The importance of satellite glia is reinforced by single cell transcriptomic analysis which reveals diverse phenotypes of glia, with transcriptomic profiles suggesting functions such as neuronal repair, regulation of neuronal metabolism, immune response and regulation of extracellular ion concentrations.^139^ In mice, while glia normally act to restrain sympathetic activity,^137^ glial activation in the stellate ganglia enhances sympathetic output to the heart, increasing heart rate and myocardial contraction and cardiac norepinephrine release.^140^ Furthermore, sympathetic nerve damage activates glia in sympathetic ganglia, providing a mechanism through which cardiac damage may trigger glial activation and stellate ganglion remodelling.^141^

Radiotherapy may be particularly well suited for neuromodulation of the stellate ganglia as glial cells seem to be more radiosensitive than neurons.^142^ Preclinical work has demonstrated that radiotherapy can inhibit satellite glial activation in dorsal root ganglia without impairing sensory or motor neuronal function.^102^ In parallel, the anti-inflammatory effects of low dose radiotherapy are well established and have been exploited therapeutically in a range of inflammatory conditions.^143^ Notably, a surprising finding from cardiac radiotherapy trials is that low dose whole heart radiotherapy improves cardiac function through anti-inflammatory effects.^144^ Furthermore, doses of approximately 25 Gy used in most cardiac radiotherapy studies to date are below the threshold associated with cardiac fibrosis. Instead, antiarrhythmic effects at these doses likely arise from alterations in cardiomyocyte gene expression, including of ion channels and gap junction proteins, thus changing their electrophysiological properties.^145 146^ Current evidence thus supports the hypothesis that subablative radiation doses may be sufficient to modulate sympathetic output through combined anti-inflammatory, glial-suppressive and transcriptional mechanisms, without causing irreversible neuronal injury. As such, radiotherapy may offer a safer alternative to surgical CSD, with the potential to minimise procedure-related morbidity while preserving therapeutic efficacy.

However, as the use of radiotherapy to the stellate ganglia for the treatment of VA is novel, both the nature and incidence of complications are unknown. Overall rates of complications reported in surgical CSD series are very variable, though off-site neurological effects are consistently the most common.^87 88 147^ Altered sensation, temperature regulation or reduced sweating in the arm or face are reported in up to 70% of patients, while acute Horner’s syndrome occurs in up to 20% of patients but persists in less than 10%.^88^ Many of these complications are at least in part due to physical manipulation or inadvertent injury to adjacent neural structures during surgery.^80 148 149^ We therefore anticipate fewer off-site neurological effects by virtue of our non-invasive approach. Furthermore, surgical complications, including pneumothorax, haemothorax and wound infections, which occur in up to 15% of patients,^87^ would be avoided entirely. Indeed, in the only published case series of radiotherapy to the stellate ganglia, three patients underwent 40 Gy stereotactic radiosurgery bilaterally to the stellate ganglia for refractory angina with no acute or periprocedural complications reported.^150^

Expected self-limiting adverse effects of cervical radiotherapy include fatigue, skin irritation and mucositis leading to cough, difficulty in swallowing or indigestion.^109 151^ Severe complications such as oesophageal fistulas or strictures are unlikely at doses below 60 Gy and the expected frequency in our study is well below 5%.^152^ Finally, stochastic effects of ionising radiation such as secondary malignancy may manifest after many years or decades. However, given the poor prognosis of patients with severe heart failure and recurrent VAs—with mortality rates of over 50% at 5 years^153^—such late effects may never become clinically apparent.

In this context, the RADIO-STAR study will provide critical first-in-human safety data and radiotherapy dose information for stellate ganglion targeting. These findings will be essential for informing the design of future larger-scale randomised trials aimed at evaluating the efficacy and durability of this novel neuromodulatory approach.

## Supplementary material

10.1136/bmjopen-2025-110958 online supplemental file 1

10.1136/bmjopen-2025-110958 online supplemental file 2

10.1136/bmjopen-2025-110958 online supplemental file 3

10.1136/bmjopen-2025-110958 online supplemental file 4
