# Supplementary material for: Protocol of the RADIO-STAR trial: a phase 1 safety and dose finding study of hypofractionated radiotherapy to the stellate ganglia for the treatment of ventricular arrhythmia
Source: BMJ Open. 2026 Feb 25;16(2):e110958. doi: 10.1136/bmjopen-2025-110958 (PMC12959014; doi:10.1136/bmjopen-2025-110958)
Supplement: online supplemental file 1 [file bmjopen-16-2-s001.pdf]

DEPARTMENT OF PHYSIOLOGY, ANATOMY AND GENETICS
Defining Excellence
Oxford Anatomy and Physiology ranked #1 in the QS World University Rankings by subject 2017, 2018

UNIVERSITY OF OXFORD

Department of Physiology, Anatomy and Genetics
Sherrington Building
Parks Road
Oxford
OX1 3PT

Full study title	Hypofractionated radiotherapy to the stellate ganglia for ventricular arrhythmia		
Participant ID			
Researchers: [redacted]		Chief Investigator: [redacted]	

Consent form

If you agree, please initial

1. I confirm that I have read and understand the information sheet (version 2.0) for the above study. I have had the opportunity to consider the information, including the risks and benefits of study participation, to ask questions and have had these answered satisfactorily	
2. I understand that my participation is voluntary and that I am free to withdraw at any time without giving any reason, without my medical care or legal rights being affected.	
3. I understand that relevant sections of my medical notes, databases of cardiovascular outcomes and data collected during the study may be looked at by researchers and authorised individuals from the Sponsor, regulatory authorities or from the NHS Trust, where it is relevant to my taking part in this research. I give permission for these individuals to have access to my records.	
4. I understand that if I choose to withdraw from the study, data already collected will be retained and used unless I request otherwise.	
5. I agree to my General Practitioner being informed of my participation in the study	
6. I agree to my General Practitioner to being contacted to provide the research team with information on my progress	
7. I agree to donate blood samples. I consider these samples a gift to the University of Oxford and I understand I will not gain any direct personal or financial benefit from them.	

Department of Physiology, Anatomy and Genetics
Sherrington Building
Parks Road
Oxford
OX1 3PT

8. I agree to take part in this study		
Optional:		
9. I would like to receive a copy of the results of this research	Yes	No
10. I agree for my samples to be used, in a form that does not identify me, in future research here or abroad, which has ethics approval. I understand this research may involve commercial organisations.	Yes	No
11. I agree to be contacted about ethically approved research studies for which I may be suitable. I understand that agreeing to be contacted does not oblige me to participate in any further studies.	Yes	No

___________________________ ____________________ ________________________________

Name of Participant *Date* *Signature*

___________________________ ____________________ ________________________________

Name of Person taking Consent *Date* *Signature*

When completed: 1 copy for participant; 1 for researcher site file (original); 1 copy to be kept in medical notes
