# Supplementary material for: Protocol of the RADIO-STAR trial: a phase 1 safety and dose finding study of hypofractionated radiotherapy to the stellate ganglia for the treatment of ventricular arrhythmia
Source: BMJ Open. 2026 Feb 25;16(2):e110958. doi: 10.1136/bmjopen-2025-110958 (PMC12959014; doi:10.1136/bmjopen-2025-110958)
Supplement: online supplemental file 2 [file bmjopen-16-2-s002.pdf]

© Copyright: The University of Oxford and Oxford University Hospitals NHS Foundation Trust 2019

Study Title: Hypofractionated RADIOtherapy to the STellate ganglia for ventricular ARrhythmia

Internal Reference Number / Short title: RADIO STAR

Ethics Ref: REC/SC/005

IRAS Project ID: 327283

Date and Version No: Version 2.1 27/6/24

Statistician Signature: Not Applicable

We declare that there are no potential conflicts of interest.

© Copyright: The University of Oxford and Oxford University Hospitals NHS Foundation Trust 2019

TABLE OF CONTENTS

1. KEY CONTACTS 6
2. LAY SUMMARY 7
3. SYNOPSIS 7
4. ABBREVIATIONS 9
5. BACKGROUND AND RATIONALE 10
6. OBJECTIVES AND OUTCOME MEASURES 15
7. STUDY DESIGN 16
8. PARTICIPANT IDENTIFICATION 19
   8.1. Study Participants 19
   8.2. Inclusion Criteria 19
   8.3. Exclusion Criteria 20
9. PROTOCOL PROCEDURES 21
   9.1. Recruitment 21
   9.2. Screening and Eligibility Assessment 21
   9.3. Informed Consent 21
   9.4. Description of study intervention(s), comparators and study procedures (clinical) 22
      9.4.1. Description of study intervention(s) 22
      9.4.2. Description of study procedure(s) 24
   9.5. Baseline Assessments 27
   9.6. Subsequent Visits 28
   9.7. Sample Handling 31
   9.8. Early Discontinuation/Withdrawal of Participants 31
   9.9. Definition of End of Study 32
10. SAFETY REPORTING 32
   10.1. Independent safety monitoring committee 32
   10.2. Definition of Serious Adverse Events 33
   10.3. Reporting Procedures for Serious Adverse Events 33
   10.4. Reporting Procedures for Device Related Adverse Events 33
   10.5. Follow-up of Serious Adverse Events 34
11. STATISTICS AND ANALYSIS 34
   11.1. Statistical Analysis Plan (SAP) 34
   11.2. Description of the Statistical Methods 34

© Copyright: The University of Oxford and Oxford University Hospitals NHS Foundation Trust 2019

11.3. Sample Size Determination 34
11.4. Analysis populations 35
11.5. Decision points 35
11.6. Stopping rules 35
11.7. The Level of Statistical Significance 35
12. DATA MANAGEMENT 35
12.1. Source Data 35
12.2. Access to Data 36
12.3. Data Recording and Record Keeping 36
13. QUALITY ASSURANCE PROCEDURES 37
13.1. Risk assessment 37
13.2. Study monitoring 37
13.3. Study Committees 37
14. PROTOCOL DEVIATIONS 37
15. SERIOUS BREACHES 38
16. ETHICAL AND REGULATORY CONSIDERATIONS 38
16.1. Declaration of Helsinki 38
16.2. Guidelines for Good Clinical Practice 38
16.3. Approvals 38
16.4. Other Ethical Considerations 38
16.4.1. Cardiac sympathetic denervation 38
16.4.2. Incidental findings 39
16.5. Reporting 40
16.6. Transparency in Research 40
16.7. Participant Confidentiality 40
16.8. Expenses and Benefits 40
17. FINANCE AND INSURANCE 40
17.1. Funding 40
17.2. Insurance 41
17.3. Contractual arrangements 41
18. PUBLICATION POLICY 41
19. DEVELOPMENT OF A NEW PRODUCT/ PROCESS OR THE GENERATION OF INTELLECTUAL PROPERTY 41
19. ARCHIVING 41
20. REFERENCES 41

© Copyright: The University of Oxford and Oxford University Hospitals NHS Foundation Trust 2019

21. APPENDIX C: AMENDMENT HISTORY 47

© Copyright: The University of Oxford and Oxford University Hospitals NHS Foundation Trust 2019

1. KEY CONTACTS

**Chief Investigator**	
**Sponsor**	University of Oxford<br>Research Governance, Ethics and Assurance (RGEA)<br>Boundary Brook House,<br>Churchill Drive, Headington,<br>Oxford, OX3 7GB<br><br>T: 01865 616480<br>E: rgea.sponsor@admin.ox.ac.uk
**Funder(s)**	British Heart foundation<br><br>[redacted]<br>Research Funds<br>British Heart Foundation<br>t: 020 7554 0434<br>e: research@bhf.org.uk
**Clinical Trials Unit**	Not applicable
**Statistician**	Not applicable
**Committees**	Independent safety committee

© Copyright: The University of Oxford and Oxford University Hospitals NHS Foundation Trust 2019

2. LAY SUMMARY

Life threatening heart rhythms are often triggered by groups of nerves that speed up the heart. For patients at high risk of these dangerous rhythms implantable cardioverter defibrillators (ICD) can prevent death by terminating such rhythms, however they cannot prevent them from occurring in the first place. Despite all currently available medical therapy many patients experience recurrent dangerous rhythms and recurrent ICD shocks, leading to worse outcomes and significant impact on quality of life. Surgically removing the nerves that trigger these dangerous rhythms is a proven treatment for such patients. However the risk of complications from such a surgery is high. Highly targeted radiotherapy using CT or MRI guidance, delivered within a limited number of treatments, allows the treatment of small cancers with very high levels of accuracy (down to a millimetre or less). This technology offers the ability to precisely target and modify the nerves going to the heart in a step wise manner without the risk of complications associated with surgery. This could potentially revolutionise treatment for patients who suffer from recurrent dangerous heart rhythms. We propose a study to establish the feasibility and safety of this approach.

3. SYNOPSIS

Study Title	Hypofractionated radiotherapy to the stellate ganglia for ventricular arrhythmia
Internal ref. no. / short title	**RADIO STAR** (hypofractionated RADIOtherapy to the STellate ganglia for ventricular ARrhythmia
Study registration	The trial will be registered on the International Traditional Medicine Clinical Trial Registry (ISRCTN).
Sponsor	University of Oxford<br>Research Governance, Ethics and Assurance (RGEA)<br>Boundary Brook House,<br>Churchill Drive, Headington,<br>Oxford, OX3 7GB<br><br>T: 01865 616480<br>E: rgea.sponsor@admin.ox.ac.uk
Funder	British Heart foundation<br>[redacted]<br>Research Funds<br>British Heart Foundation<br>t: 020 7554 0434<br>e: research@bhf.org.uk
Study Design	Case series
Study Participants	Patients with structural heart disease and MR-conditional Implantable Cardioverter Defibrillators who have received more than one appropriate therapy (shock or anti-tachycardia pacing) for ventricular arrythmia

© Copyright: The University of Oxford and Oxford University Hospitals NHS Foundation Trust 2019

Sample Size	13 patients		
Planned Study Period	4 years (May 2024- May 2028)		
Planned Recruitment period	May 2024- May 2028		
	Objectives	Outcome Measures	Timepoint(s)
Primary	Assess the safety of radiotherapy to the stellate ganglia in patients with recurrent ventricular arrythmia	Treatment related serious adverse events (SAEs) defined as any grade 3 toxicity requiring hospitalization or any grade 4 to 5 toxicity in the first 6 months as defined by the common terminology criteria for adverse events	During all radiotherapy sessions and monitoring visits up to 6 months post radiotherapy
		Treatment related side effects based on patient symptom questionnaires and clinical examination during study visits	During all radiotherapy sessions and monitoring visits up to 6 months post radiotherapy
Secondary	Assess the feasibility of radiotherapy to modify the stellate ganglia to achieve anatomical and functional sympathetic denervation.	Physical modification of the stellate ganglia as assessed on MRI imaging	MRI scan at the final study visit 6 months post radiotherapy
		Haemodynamic evidence of stellate ganglia modification determined by clinical and device-based heart rate dynamics and variability	Monitoring visits up to 6 months post radiotherapy
		Changes in circulating biomarker levels	Monitoring visits up to 6 months post radiotherapy
		The number of ventricular arrhythmias requiring device therapy	Monitoring visits up to 6 months post radiotherapy
		Patient reported outcomes about quality of life and symptoms using the KCCQ-23 questionnaire	Monitoring visits up to 6 months post radiotherapy
	Correlate the change in circulating	Changes in serum biomarker levels prior to compared to following	During initial visit and monitoring visits up to

© Copyright: The University of Oxford and Oxford University Hospitals NHS Foundation Trust 2019

	biomarker levels with the efficacy of radiotherapy to modify the stellate ganglia in terms of arrhythmic burden	intervention. Reduction in the number of ICD shocks/ATP in the 6 months before compared to the 6 months after treatment	6 months post radiotherapy
Intervention(s)	Hypofractionated radiotherapy to the stellate ganglia		
Comparator	There is no control group		

4. ABBREVIATIONS

ATP	Anti-tachycardia Pacing
BHF	British Heart Foundation
CI	Chief Investigator
CIED	Cardiac Implantable Electronic Device
CMR	Cardiac Magnetic Resonance
CRF	Case Report Form
CRT-D	Cardiac Resynchronization Therapy with ICD
CSD	Cardiac Sympathetic Denervation
CT	Computed tomography
CTCAE	Common Terminology Criteria for Adverse Events
DAD	delayed afterdepolarisations
DLT	Dose Limiting Toxicity
ECG	Electrocardiograph
eGFR	Estimated Glomerular Filtration Rate
GCP	Good Clinical Practice
HFrEF	Heart Failure with Reduced Ejection Fraction
ICD	Implantable Cardioverter Defibrillator
ICF	Informed Consent Form
ICNS	Intrinsic Cardiac Nervous System
MRI	Magnetic Resonance Imaging
MR-Linac	Magnetic Resonance Linear Accelerator
NHS	National Health Service

© Copyright: The University of Oxford and Oxford University Hospitals NHS Foundation Trust 2019

NPY	Neuropeptide-Y
NRES	National Research Ethics Service
OCMR	University of Oxford Centre for Clinical Magnetic Resonance Research
OUHFT	Oxford University Hospitals NHS Foundation Trust
PI	Principal Investigator
PIL	Participant/ Patient Information Leaflet
PROM	Patient Reported Outcome Measures
R&D	NHS Trust Research & Development Department
REC	Research Ethics Committee
RNAseq	Ribonucleic acid sequencing
SAE	Serious Adverse Event
SCD	Sudden Cardiac Death
SOP	Standard Operating Procedure
STEMI	ST-Elevation Myocardial Infarction
STIR	short-*tau* inverted recovery
Tp-e	electrocardiographic T wave
True-FISP	True fast imaging with steady state precession
UCLA	University of California, Los Angeles
UK	United Kingdom
VA	Ventricular Arrhythmia
VATS	Video-assisted thoracoscopic surgery

5. BACKGROUND AND RATIONALE

a) Background to the Study and Pilot Data

Background

Ventricular arrhythmias (VA) are a life-threatening event and are the most common cause of cardiac arrest and sudden cardiac death (SCD) (1). It is estimated that SCD accounts for up to 20% of deaths in the western world(2). Patients with structural heart disease such a heart failure with reduced ejection fraction (HFrEF) are at increased risk of SCD. There are around 900,000 people living with heart failure in the UK (3) with an estimated 200,000 new diagnoses each year(4). Despite advances in medical therapy, HFrEF is still associated with a high mortality rate, a large proportion of which is due to SCD(5).

To-date beta-blockers are the only primary prevention anti-arrhythmic drugs that are proven to reduce mortality due to VAs in heart failure patients (5, 6). Other classes of antiarrhythmic drugs lack a mortality

© Copyright: The University of Oxford and Oxford University Hospitals NHS Foundation Trust 2019

benefit(7-9) or even lead to excess mortality through pro-arrhythmic effects(10). However, despite optimal medical therapy including beta-blockers, the risk of arrhythmia and death in HFrEF remains high, at around 7-10% annually(9, 11). In patients at very high risk, such as those with severe heart failure or who have survived a VA event, the implantable cardioverted defibrillator (ICD) improves mortality(9)(11).While such devices are effective at treating VAs once they occur, and thus reducing mortality associated with SCD, they do not prevent the arrhythmia occurring in the first place. Patients with incessant VA's may thus experience recurrent device therapy associated with increased mortality(12) and significant impact on quality of life. Prevention of VAs is thus vital. Anti-arrhythmic drugs can reduce VA episodes but are associated with side-effects and limited efficacy(13) . While catheter ablation has been shown to be effective at reducing VAs(14) a significant number of patients continue to have refractory VAs, and the main large trials of VT ablation (eg SMASH-VT, VTACH, SMS, VANISH) have failed to demonstrate a significant reduction in mortality which remains high(15-18). Few remaining options therefore exist for such patients. More recently, surgically removing the sympathetic nerves that innervate the heart via removal of the stellate ganglia (cardiac sympathetic denervation) has proven to be an effective treatment(19, 20) although surgical complication rates are high(19).

The sympathetic nervous system and arrhythmia

Most VAs are triggered by the sympathetic nervous system(21), hence the protective effects seen with beta-blockade. VAs require both a trigger and appropriate substrate to result in sustained arrhythmia. Nor-adrenaline and sympathetic co-transmitters such as NPY influence the electrophysiological properties of the heart to predispose to this. Beta-adrenergic stimulation increases intracellular calcium loading in myocytes, predisposing to delayed afterdepolarisations (DAD) which are triggers for most pathological arrhythmias(22-24). Indeed increasing sympathetic activation to the heart through stellate ganglion stimulation has been shown to induce DADs *in vivo*(25). For a DAD to trigger an arrhythmia it must encounter an appropriate substrate to form a self-sustaining circuit, usually in the form of re-entry around an area of anatomical or functional block. In general, slow conduction velocities and shorter action potential durations favour the formation of sustainable re-entry circuits as they allow faster myocardial recovery from previous depolarisation to sustain a circuit. Beta-adrenergic stimulation in known to increase I_{KS} (slow outward potassium current) which results in shorter action potential duration(26). Furthermore, due to regional differences in sympathetic innervation, sympathetic activation has been shown to produce heterogeneity in cardiac myocyte electrical properties in the heart (27) which predisposes to arrhythmia. It has been demonstrated *in vivo* that stellate ganglion stimulation increases the time interval from the peak to the end of the electrocardiographic T wave (Tp-e), which considered a marker of dispersion of repolarisation in the heart, and is an independent predictor for risk of SCD(28).

Pre-ganglionic sympathetic fibres to the heart arise from the intermediolateral column of the spinal cord at the level of T1-T6. They exit the spinal cord via the ventral nerve roots and project towards post-synaptic efferent fibres contained in the paravertebral sympathetic chain. Most cardiac post-synaptic sympathetic fibres arise in the stellate ganglion; a fusion of the lower cervical and first thoracic ganglia(29, 30). It is being increasingly recognised that autonomic control of the heart involves a complex cardio-neural hierarchy comprising of the central nervous system, thoracic ganglia and intrinsic cardia neurons which form the intrinsic cardiac nervous system (ICNS)(21). Within this system sympathetic co-transmitters such as NPY also play an important role in mediating sympathetic effects on the heart (31, 32).

© Copyright: The University of Oxford and Oxford University Hospitals NHS Foundation Trust 2019

Cardiac sympathetic denervation

Given the complexity of autonomic control of the heart and the effects of sympathetic co-transmitters such as NPY, beta-adrenergic receptor blockade likely only affects a small piece of a much larger puzzle when considering neuro-modulation of the heart. It is thus unsurprising that many patients continue to have VA's despite maximal betablocker therapy. This limitation has led to the introduction of other ways to achieve neuromodulatory control of the heart.

Selective modulation of sympathetic efferents to the heart has shown promising antiarrhythmic effects and offers a number of theoretical benefits over medical therapy. Selectively targeting cardiac sympathetic fibres prevents systemic side effects associated with beta-blocker therapy, and would provide a more comprehensive reduction in sympathetic effects on the heart.

Reducing sympathetic tone through deep sedation has been shown to be effective in reducing VAs in patient's refractory to antiarrhythmic medication (33). Percutaneous injection of local anaesthetic into the epidural space at level T1-T4 can be used to temporarily reduce sympathetic outflow to the heart. This technique, called thoracic epidural anaesthesia (TEA), has been successfully used to manage refractory arrhythmias in patients with structural heart disease (34) as just a bridge to more definitive therapy (35).

A more permanent form of cardiac sympathetic denervation (CSD) can be achieved through surgical removal of the lower half of the stellate ganglia along with the T2-T4 sympathetic ganglia(34, 36), done as a video assisted thoracoscopy (VATs) procedure. This has been shown to effectively reduce VA burden in patients with long QT syndrome (37), catecholaminergic polymorphic ventricular tachycardia(38) and patients with structural heart disease (20). Indeed, a study of 121 patients with severe heart failure and refractory VAs, showed CSD resulted in a >80% reduction in ICD therapy (36). Overall bilateral CSD seems to be more effective than left-only CSD(20).

Unfortunately, this procedure also carries a high complication rate. In a systematic review, including 173 patients, complications occurred in 34% of patients.(19).Surgical complications include pneumothorax, haemothorax and wound complications, with reported frequency up to 15% (19, 39, 40). Even more commonly, in up to 30% of patients, off-site neurological effects such as upper limb neuropathic pain, altered sweating, Horner's syndrome and transient hypotension occur. These are however generally mild and transient(19, 41), and importantly even when they do persist don't affect patients' quality of life (42, 43). Interestingly it seems that many of the off-site neurological complications result from the physical manipulation and damage of nerves during surgery. Furthermore, recent evidence suggests that the risk of Horner's syndrome and hyperalgesia may be mitigated by sparing the cranial portion of the stellate ganglion, and the T3-T4 paravertebral ganglia whilst still achieving sufficient CSD(30).

We thus hypothesise that a non-invasive technique, targeting the lower half of the stellate ganglia bilaterally, aiming for modification rather than complete destruction would provide a more favourable complication profile whilst maintaining anti-arrhythmic efficacy. Indeed, recently Nonoguchi et al. described transiently achieving successful CSD using phototherapy of the stellate ganglion without complications(44).

Hypofractionated radiotherapy for cardiac sympathetic denervation

© Copyright: The University of Oxford and Oxford University Hospitals NHS Foundation Trust 2019

Improvements in radiotherapy planning and delivery now allow larger, highly targeted radiotherapy treatments to be delivered over a few days. . This allows precise delivery of radiation to targets with rapid dose fall-off to minimise any damage to surrounding normal tissue (45-47). We propose to use hypofractionated radiotherapy to target the stellate ganglia to achieve modification in a non-invasive manner.

With regards to MRI based radiotherapy treatment delivery, historically there have been safety concerns regarding the use of MRI in patients with implanted cardiac electrical devices (CIEDs). However, there is now extensive evidence that this can be safely done if appropriate protocols are followed(48). Indeed, there is also already extensive experience and protocols available for the use of MR-Linac in patients with CIEDs(49, 50). There is also growing experience in the use of radiotherapy directed at the heart itself to treat patients with refractory ventricular arrhythmia, which has been shown to be both safe and effective (50, 51). There are now multiple cardiac radiotherapy VT ablation trials which are in the recruitment phase. MR-guided radiotherapy has also been successfully used to perform cardiac ablations in patients with implantable cardiac devices in situ(52, 53).

The use of radiotherapy for the treatment of benign lesions is well established and the Royal College of Oncologists has published recommendations for the use of radiotherapy for benign disease in the UK(54). There are already active clinical trials investigating radiotherapy directly to the heart for the treatment of dangerous heart rhythm(55), including in the SABRE-VT trial in the UK. Radiotherapy delivered directly to the heart, whilst potentially effective, is a complex undertaking requiring the integration of multiple investigations performed on different platforms to define a target. This is followed by treatment delivery to a mobile structure, the heart, with both cardiac and respiratory motion to be accounted for. This usually requires additional treatment margins to the target to account for uncertainties, thus increasing the volume of heart muscle treated in patients with already limited baseline heart function. We believe that hypofractionated radiotherapy to the stellate ganglia, rather than targeting the heart itself, may allow an alternative approach to the management of ventricular arrythmias in this treatment refractory patient group. Indeed CT-guided radiotherapy to the stellate ganglia has already been successfully been used to treat angina in a case report (56).

The radiation dose required to modify the function of the stellate ganglion has not been previously defined. The recognised safe upper limit for radiation dose to neuronal structures, e.g. brachial plexus, is 24 Gy in 3 fractions on alternate days to a point dose (0.1cc of the structure). Doses above this level may be associated with reduced neuronal function (57). This has been taken as the lower limit of radiation doses considered in this study. Using the historical literature targeting the other ganglia, e.g. trigeminal ganglia and neuronal tissue (56, 58-62) and radiobiological calculations (personal communication Professor Rob Glynn-Jones), we derived an upper limit 33 Gy in 3 fractions on alternate days. For example, the 'neurosurgical approach' used in trigeminal neuralgia and case report targeting the stellate ganglia(56), with a single very large treatment dose, is likely to be far higher than that required. Stellate ganglion modification rather than destruction may be adequate, reducing the risk of neurological sequalae seen with surgical excision and allows the radiation dose to nearby organs at risk (such as the brachial plexus) and the device itself to remain within low-risk limits set by national guidelines. This dose range is within that routinely used for treatments for cancer(57). Furthermore, radiation in a stepwise fashion would allow for monitoring and mitigation of any neurological side effects.

Pilot work on healthy volunteers has allowed optimisation of MRI imaging sequences, both on a diagnostic MRI 1.5T scanner and on a 0.35T MR-Linac, providing confidence in visualisation of stellate

© Copyright: The University of Oxford and Oxford University Hospitals NHS Foundation Trust 2019

ganglia and suppression of any significant imaging artefact from ICD. This has allowed planning of radiotherapy to confirm the feasibility of hypofractionated radiotherapy to target the stellate ganglia. Figure 1 below shows a 1.5T screening scan, together with a 0.35T planning scan and subsequent radiotherapy dose calculations on a subject with a left sided cardiac resynchronisation therapy defibrillator (CRTD) from which the most artefact is generated. The doses to adjacent organs at risk (see Key and 1B and C) and to the device itself (0.4 Gy) are well below their tolerance constraints according to the UK Consensus (63) and American Association of Physicists in Medicine (AAPM) guidelines (64).

Figure 1.

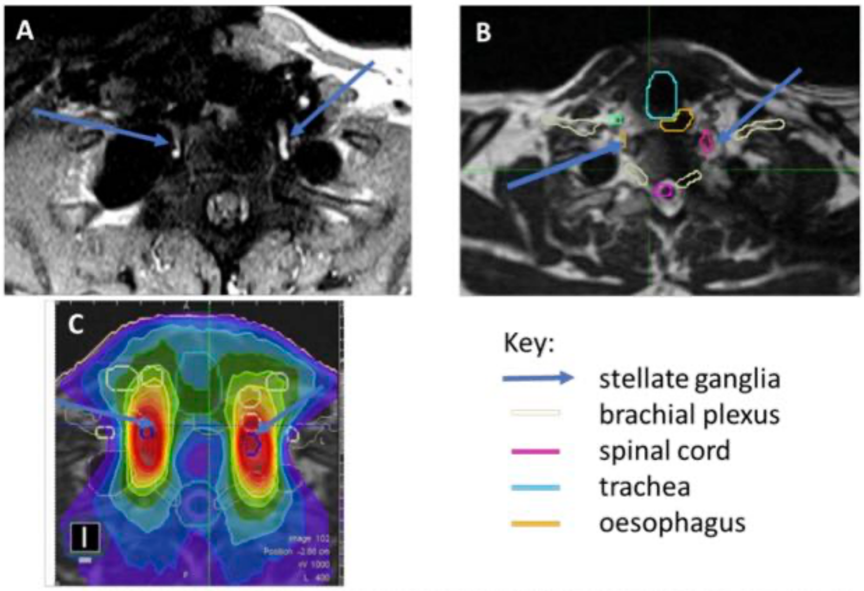

A: 1.5T T2 weighted short-*tau* inverted recovery (STIR) MR transverse image from a subject with a left sided cardiac resynchronisation therapy defibrillator (Medtronic Compia MRI™ CRT-D SureScan™). B: MR linac 0.35T True-FISP scan with treatment planning contours from the same subject. C: MR linac 0.35T True fast imaging with steady state precession (True-FISP) scan in the same subject with radiotherapy treatment isodoses; high dose levels (red) over stellate ganglion bilaterally, with rapid dose drop off and predominantly lower isodose levels (green, blue, purple) over adjacent dose sensitive structures e.g. brachial plexus, spinal cord, trachea

b) <u>Research Questions for this Study (in Lay language)</u>

Can high precision radiotherapy suppress the function of the collection of nerves that cause dangerous heart rhythms?

Does this form of high precision radiotherapy have a favourable safety profile compared to alternative treatments (e.g. medications, cardiac ablation or surgical removal)?

Does high precision radiotherapy affect levels of circulating markers in blood samples known to increase risk of dangerous heart rhythms?

Does high precision radiotherapy result in a reduction in abnormal heart rhythms and is this related to any changes in the levels of markers in blood samples?

© Copyright: The University of Oxford and Oxford University Hospitals NHS Foundation Trust 2019

6. OBJECTIVES AND OUTCOME MEASURES

Objectives	Outcome Measures	Timepoint(s) of evaluation of this outcome measure (if applicable)
**Primary objectives**		
Assess the safety of hypofractionated radiotherapy to modify the stellate ganglia in patients with recurrent ventricular arrythmia	Treatment related serious adverse events (SAEs) defined as any grade 3 toxicity requiring hospitalization or any grade 4 to 5 toxicity in the first 6 months as defined by the common terminology criteria for adverse events	During all hypofractionated radiotherapy sessions and monitoring visits up to 6 months post radiotherapy
	Treatment related side effects based on patient symptom questionnaires and clinical examination during study visits	During all hypofractionated radiotherapy sessions and monitoring visits up to 6 months post radiotherapy
**Secondary objectives**		
Assess the feasibility of hypofractionated radiotherapy to modify the stellate ganglia to achieve anatomical and functional sympathetic denervation.	Physical modification of the stellate ganglia as assessed on MRI imaging	MRI scan at the final study visit 6 months post radiotherapy
	Haemodynamic evidence of stellate ganglia modification determined by clinical and device-based heart rate dynamics and variability	Monitoring visits up to 6 months post radiotherapy
	Changes in circulating biomarker levels	Monitoring visits up to 6 months post radiotherapy
	The number of ventricular arrhythmias requiring device therapy	Monitoring visits up to 6 months post radiotherapy
	Patient reported outcomes about quality of life and symptoms based on KCCQ questionnaire	Monitoring visits up to 6 months post radiotherapy

© Copyright: The University of Oxford and Oxford University Hospitals NHS Foundation Trust 2019

Correlate the change in circulating biomarker levels with the efficacy of hypofractionated radiotherapy to modify the stellate ganglia in terms of arrhythmic burden	Changes in serum biomarker levels prior to compared to following intervention. Reduction in the number of ICD shocks/ATP in the 6 months before compared to the 6 months after treatment	During initial visit and monitoring visits up to 6 months post radiotherapy

7. STUDY DESIGN

Our study is a proof-of-concept case series to assess the safety and feasibility of non-invasive stellate ganglia modification achieved with MR-guided radiotherapy in patients who have experienced life threatening ventricular arrythmias. We will recruit patients with structural heart disease and impaired left ventricular systolic function who are already on optimum medical therapy and have MR-conditional ICDs (implanted at least 6 months ago), who have recently required appropriate ICD therapy (> 1 shock or ATP) for ventricular arrhythmia.

© Copyright: The University of Oxford and Oxford University Hospitals NHS Foundation Trust 2019

All participants will initially undergo a 1.5T MRI scan at OCMR obtain baseline images of the stellate ganglia, and to assess feasibility of hypofractionated radiotherapy to target the stallage ganglia (ability to clearly visualise the stellate ganglia, size of the stellate ganglia and their proximity of nearby organs at risk, and ability to tolerate MRI scanning). Once confirmed that the patient would be suitable for treatment, they would have an appointment with our collaborating oncologist colleagues to have dedicated planning scans. A radiotherapy treatment plan will be developed based on the scan data with the aim to deliver 3 fractions of treatment on alternate days to the lower half of the stellate ganglia and T1-2 sympathetic chain. The radiotherapy planning process will include determining the radiation doses delivered to the ICD device and nearby organs at risk. If it is not possible to safely deliver radiotherapy without exceeding the limit for low-risk radiotherapy (as defined by the UK Consensus and AAPM 2019) the patient will be withdrawn from the trial as detailed in section 9.8.

A dose escalation protocol will be used to determine the lowest radiotherapy dose that can safely achieve sympathetic downregulation. This protocol will involve 13 patients with the doses escalated as follows: The first 3 patients would be treated at 8 Gy per fraction for 3 fractions on alternate days. If no serious adverse events occur then we will increase the dose to 9 Gy per fraction for 3 fractions on alternate days in the next 3 patients, 10 Gy per fraction for the next 3 patients for 3 fractions on alternate days and 11 Gy per fraction for 3 fractions on alternate days for the final 4 patients (figure 2). There will be a minimum of 6 weeks between the last participant treated at each dose before escalation the next dose to allow for detection of adverse events at participants' 6 week follow up visit. Before each dose escalation we will have an independent safety committee review all patient data and radiotherapy doses will only be escalated if there are no safety concerns.

Our study is a safety study, and we are powered to detect serious adverse events as our primary endpoint. Serious adverse events (SAEs) defined as any related and unexpected grade 3 toxicity requiring hospitalization, grade 4 toxicity or grade 5 toxicity as defined by the common terminology criteria for adverse events.

© Copyright: The University of Oxford and Oxford University Hospitals NHS Foundation Trust 2019

If a serious adverse event occurs, then no further fractions would be given to that patient and the dose would be reduced by 1 Gy for all subsequent patients (up to a total of 13 patients) without further dose escalation. If an SAE occurs at the lowest radiotherapy dose (8 Gy per fraction for 3 fractions) all subsequent patients will receive treatment at the even lover dose of 7 Gy per fraction for 3 fractions, if approved by our independent safety committee. However as highlighted in section 5 we have chosen a starting dose within the safe upper limit for neuronal tissue, so we do not anticipate this. An early stopping rule will be set to halt enrolment if 4 out of the first 8 patients develop and SAE. If patients withdraw from this trial then they will be replaced, and the study will continue until 13 patients have completed the dose escalation protocol (as detailed in section 9.8). We will not recruit any extra participants into our dose escalation protocol, unless participants withdraw, in which case they will be replaced. Once 13 participants complete the dose escalation protocol no further patients will be recruited.

Figure 2.

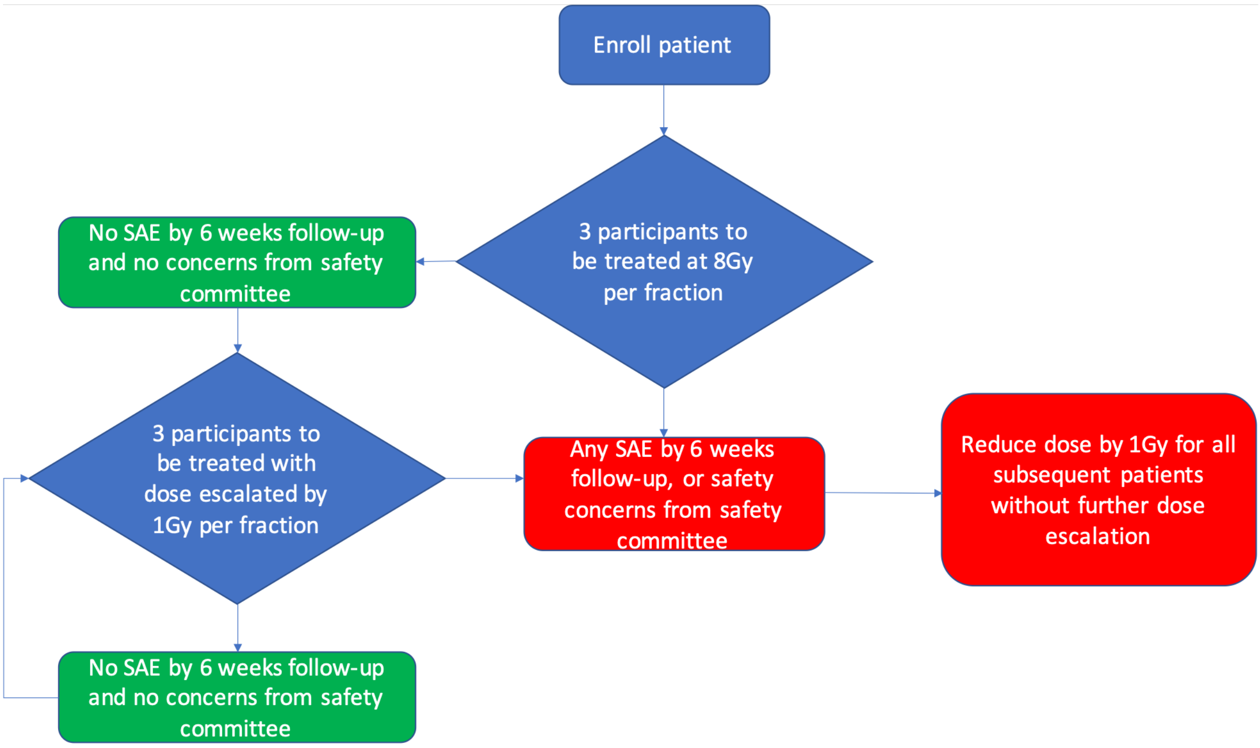

Figure 2. The primary safety endpoints of this trial is any related and unexpected SAE occurring. SAE is defined in this trial as any Grade 5, Grade 4 or Grade 3 toxicity requiring hospitalisation as per the Common terminology criteria for adverse events (CTCAE v5.0). The first 3 patients will be treated at 8 Gy per fraction for 3 fractions on alternate days to achieve a total dose of 24 Gy. If no SAE is detected in any patient by 6 weeks, then the dose will be escalated by 1Gy per fraction for the subsequent 3 patients. This will be repeated up to a maximum dose of 11Gy per fractions (total 33Gy) for the final 4 patients. If any SAEs are detected then no further fractions of radiotherapy will be given to that patient, and the dose would be reduced

© Copyright: The University of Oxford and Oxford University Hospitals NHS Foundation Trust 2019

by 1Gy per fraction for all subsequent patients without further dose escalation during the trial. An early stopping rule will be set to halt protocol enrolment if ≥4 of the first 8 patients developed an SAE.

8. PARTICIPANT IDENTIFICATION

8.1. Study Participants

We aim to recruit thirteen adult participants with structural heart disease and impaired LV systolic function who have a primary or secondary prevention MR-conditional ICD device and have received more than one appropriate shock or ATP therapy in the preceding 6 months. We will recruit patients who receive care from cardiology services of the Oxford University Hospitals NHS Foundation Trust. They should have had their ICD *in-situ* for a minimum of 6 months and should be on optimal guideline based medical therapy for heart failure at the time of enrolment. We would look to screen at least 16 patients to allow for a 20% drop out rate. Eligible patients will be invited to undergo a screening 1.5T MRI scan at OCMR to and to assess the feasibility of hypofractionated radiotherapy to target the stallage ganglia (ability to clearly visualise the stellate ganglia, size of the stellate ganglia and their proximity of nearby organs at risk, and ability to tolerate MRI scanning) and to provide a baseline imaging from which to assess for physical modification to the stellate ganglia after 6 months. Once confirmed that the patient would be suitable for hypofractionated radiotherapy treatment, they would have an appointment with our collaborating oncologists to have dedicated planning scans.

We implant over 150 ICDs per year at Oxford University Hospitals NHS Foundation Trust and currently have over 500 ICD patients set up on remote monitoring. We also perform between 50 and 75 VT ablations per year in this population most of whom would be eligible for recruitment. Identifying 13 patients with appropriate therapies via remote monitoring, device clinics and acute admissions (as a tertiary centre) should therefore easily be achieved over a 24-month period.

Heart failure participants are likely to be taking multiple medication including (but not limited to) ACE inhibitors, combined angiotensin receptor/neprilysin inhibitors, beta blockers, SGLT2 inhibitors and aldosterone antagonists, however none of the items in the study protocol will impact upon routine heart failure medications.

8.2. Inclusion Criteria

General Inclusion Criteria

© Copyright: The University of Oxford and Oxford University Hospitals NHS Foundation Trust 2019

- Participant is willing and able to give informed consent for participation in the study.
- Male or Female, aged at least 18 years old.
- Known diagnosis of structural heart disease defined as any patient with impaired left ventricular ejection fraction (less than 55%) due to any cause including ischaemic cardiomyopathy, dilated cardiomyopathy, hypertrophic cardiomyopathy or Arrhythmogenic cardiomyopathy.
- CMR compatible ICD device implanted a minimum of 6 months ago, under follow-up at_Oxford University Hospitals NHS Foundation Trust.
- Experienced more than one appropriate ICD therapy (shocks or ATP) for ventricular arrhythmia in the last 6 months
- Established on optimal guideline based medical therapy for heart failure and arrhythmia

8.3. Exclusion Criteria

General Exclusion Criteria

- Device radiation dose or nearby organs at risk exceeding the limit for low-risk radiotherapy as defined by the UK Consensus and AAPM 2019 guidelines
- Severe kidney impairment (eGFR less than 30) which would prevent the safe use of iodine contrast during CT scans
- Female patients who are pregnant, lactating or planning pregnancy during the study period
- Patients who are terminally ill, inappropriate for intervention, or unable to consent
- Any impediment to communication which, in the opinion of the investigator, might prevent the investigator communicating effectively with the patient during the study which could cause a safety or reliability concern.
- Any other condition which, in the opinion of the investigator, might affect the safety of the participant or reduce the reliability of the study results
- Involvement in any other research project where the procedures would affect the outcomes of this study.

MRI safety exclusion criteria

- Metal clips or metallic foreign body
- Prior injury to the eye involving fragments of metal
- Prior shrapnel injuries
- Any other metallic or electronic implants affected by the magnetic field
- History of severe claustrophobia
- Severe liver damage, TB, pulmonary disease, anaemia, blood coagulation disorders

© Copyright: The University of Oxford and Oxford University Hospitals NHS Foundation Trust 2019

9. PROTOCOL PROCEDURES

9.1. Recruitment

Patients attending outpatient heart failure or arrhythmia clinics, under existing follow-up in pacing clinic, or admitted as an in-patient under the electrophysiology team will be identified by their routine clinical care team. Either a letter of invitation along with the Patient information leaflet (PIL) will be sent ahead of their clinical appointment along with the appointment letter, or the clinical team will inform the patient on the day of their clinical appointment (a routine appointment in the heart failure clinic or in the in the arrhythmia/pacing clinic, or during their in-patient admission) about the study. Potential participants who express an interest in participation will be offered the opportunity to discuss the study with the study team and with their agreement, their contact details can be provided by the clinical team to the study team. Potential participants will be allowed at least 24 hours to consider the written and verbal study information before entering the study. The informed consent document will be signed at the first study visit by the participant and delegated study investigator.

9.2. Screening and Eligibility Assessment

As outlined above, potential participants will be identified by their routine clinical care team who will provide a copy of the PIL to patients. Patients who express interest in taking part in the study (either by directly contacting the study team, or by consenting for their clinical team to securely pass their details to the study team) will invited to a screening visit with the study team. At the screening visit the study team will formally review patients' eligibility to join the study and confirm that they are safe to continue to undergo imaging MRI assessments. Prior to any screening assessments patients will undergo an informed written screening consent process (see section 9.3). Patients will then undergo a 1.5T cardiac MRI scan to assess for any device related artefact that would prohibit successful hypofractionated radiotherapy to the stellate ganglia. Those who are confirmed eligible will then provide full consent to participate in the study proper.

9.3. Informed Consent

Participant must personally sign and date the latest approved version of the Informed Consent form before any study specific procedures are performed.

Written and verbal versions of the Participant Information and Informed Consent will be presented to the participants detailing no less than: the exact nature of the study; what it will involve for the participant; the implications and constraints of the protocol; the known side effects and any risks

© Copyright: The University of Oxford and Oxford University Hospitals NHS Foundation Trust 2019

involved in taking part. It will be clearly stated that the participant is free to withdraw from the study at any time for any reason without prejudice to future care, without affecting their legal rights, and with no obligation to give the reason for withdrawal.

Screening consent: The participant will be allowed as much time as wished to consider the information, and the opportunity to question the Investigator, their GP or other independent parties to decide whether they will participate in the study. Prior to any screening assessments or tests, a screening consent will then be obtained by means of participant-dated signature and dated signature of the person who presented and obtained the Informed Consent. The person who obtains the consent must be suitably qualified and experienced and have been authorised to do so by the Chief/Principal Investigator. A copy of the signed Informed Consent will be given to the participant and one placed in their medical notes. The original signed form will be retained at the study site. We anticipate a 20-30% screening failure so we will not proceed with formal study consent until eligibility is confirmed following screening assessment.

We will have a separate and more detailed consent process for patients who pass screening and are formally enrolled in this study. As detailed in section 9.6 of the protocol, patients will be counselled about the process and potential risks of radiotherapy by the oncology team who will perform the radiotherapy. This is to ensure that patients have had every opportunity to fully address any concerns ahead of consent to participate in a study involving radiotherapy. We believe a dedicated visit with the radiotherapy team who will explain the treatment process in detail is important, to ensure patients have fully understood the process and any potential risks involved.

9.4. Description of study intervention(s), comparators and study procedures (clinical)

9.4.1. Description of study intervention(s)

Radiotherapy

Modern radiotherapy is inherently delivered under image guidance. For this study we will have access to both CT and MRI based image guidance radiotherapy platforms, with comparable delivery accuracy on either platform. Oxford University Hospitals NHS Foundation Trust has an established on-site CT based radiotherapy service as well as access to MR-Linac based radiotherapy through service level agreements with GenesisCare.

CT based radiotherapy at the Oxford University Hospitals NHS Foundation Trust is delivered on a Varian TrueBeam™ system which combines cone beam computed tomography (CBCT) with a linear accelerator to deliver highly accurate radiotherapy. MR- based radiotherapy at GenesisCare is delivered on the MR-Linac platform. This combines 0.35T MRI imaging with a linear accelerator to again provide highly accurate radiotherapy. ..

For patients treated on the Varian TrueBeam, a conebeam CT scan will be taken prior to each radiotherapy treatment to ensure the patient is appropriately aligned to deliver treatment accurately. The

© Copyright: The University of Oxford and Oxford University Hospitals NHS Foundation Trust 2019

additional ionising radiation dose delivered during these scans is negligible compared to the ionising radiation dose delivered with each radiotherapy treatment.

The total radiation dose to the stellate ganglia is 24-33 Gy over the course of 3 fractions of treatment on alternate days. This radiation dose is targeted bilaterally to the lower half of the stellate ganglia and T1-2 paravertebral chain, with a rapid drop of to lower radiation isodose levels over adjacent dose sensitive structures such as the brachial plexus, spinal cord and trachea. Treatment will be planned while observing the organs at risk constraints i.e. acceptable limits of dose to the organ (57) and ensuring the dose to the ICD is within the threshold defined by the UK Royal College of Radiologists (63) and American Association of Physicists in Medicine (64).

Hypofractionated radiotherapy to the neck area is well tolerated (46, 65, 66) but may be associated with side effects related to the target and adjacent structures. Side effects from treatment of the stellate ganglion may result in side effects for cardiac sympathetic denervation, as listed below, including less commonly (<10%) hypotension, Horner's syndrome and upper limb and face hyperalgesia and hypohidrosis.

Other radiotherapy related side effects may include temporary mild tiredness, discomfort swallowing and a cough, less commonly a localised skin reaction, hair loss at the base of the neck/upper body. In the long term, there is a low risk of tracheo-oesophageal fistula, oesophageal narrowing, and brachial plexus injury resulting in pain, numbness and tingling sensation in the arms.

Total radiotherapy dose ranges from 24-33Gy in 3 treatment fractions depending on study cohort. Radiotherapy is associated with a multiple potential treatment side-effects. In the long term, ionising radiation can also cause cancer which manifests itself after many years or decades. The risk of developing cancer as a consequence of radiotherapy treatment is an accepted side-effect of the treatment and must be balanced against the potential overall benefit of receiving radiotherapy. However, the risk of cancer induction over several years or decades should be viewed in the context of our population life expectancy. Out study population of patients with severe heart failure and recurrent ventricular arrhythmias has a particularly poor prognosis, even despite all current available medical therapies, with mortally rates observed in excess of 50% at 5 years(67).

Cardiac sympathetic denervation

© Copyright: The University of Oxford and Oxford University Hospitals NHS Foundation Trust 2019

The aim of delivering radiotherapy to the stellate ganglion in this study is to achieve functional sympathetic denervation of the heart. Reports about the type and frequency of complications arising from cardiac sympathetic denervation come from case series of surgical removal of the stellate ganglion. A large number of complications associated this with are surgical complications such as pneumothorax, pleural effusion and infection, which do not apply to our study, and indeed is part of the motivation for this study to achieve CSD in a non-invasive manner.

The primary non-surgical complications associated with CSD are off site neurological effects and hypotension. A recent systematic review including 173 patients who had surgical CSD estimated the risk of hypotension as 9%, and off-site neurological effects as follows: Horner's syndrome 3%, and upper limb and face hyperalgesia 4% and hypohidrosis 3%(19). No procedural deaths were reported. The risk of Horner's syndrome can be mitigated by sparing the cranial portion of the stellate ganglion and sparing the T3-T4 paravertebral ganglia may reduce off site neurological complications while still achieving sufficient CSD(30). Overall experience from surgical removal of the stellate ganglia suggests that off-site neurological side effects generally mild and transient(19, 41), and importantly even when they do persist don't significantly affect patients' quality of life (42, 43).

Our study is designed to minimise the risk of complications. We will only target the lower half of the stellate ganglia and T1-2 paravertebral chain. Furthermore, by achieving CSD in a staged and stepwise manner over 3 fractions of radiotherapy we aim to detect any complications early before permanent CSD is achieved, thus mitigating the severity of these complications. Indeed, one of the co-primary outcomes of this study is any treatment related adverse effects.

9.4.2. Description of study procedure(s)

<u>**Cannulation and phlebotomy**</u>

Risk to Participants: Common risks associated with phlebotomy or cannula insertion are pain during the procedure and bruising (with associated pain afterwards). These risks will be minimised by ensuring that all staff are fully trained in phlebotomy. Bruising after the event will also be reduced by promptly applying pressure on the puncture site after the needle/cannula is withdrawn. All participants will be fully informed about these risks in the Participant Information Sheet. The worry associated with taking blood may cause some participants to feel unwell or faint before, during or after the procedure. The risk associated with this will be reduced by having an adequately equipped facility for performing the procedure (see above) and having a staff member trained in basic life support. Although cannulation is a very safe procedure, it does create a puncture wound on the skin which may very rarely lead to infection around the puncture site. The risk of this will be minimised by ensuring strict hygiene during the procedure and by not recruiting

© Copyright: The University of Oxford and Oxford University Hospitals NHS Foundation Trust 2019

participants who are at increased risk of infection. If a participant reports symptoms of an infection (local redness, swelling, pain or discharge of pus) they should be referred to their GP or to A+E urgently.

Risk to Researchers/Other Staff: Taking blood carries a risk of needle stick injury to the phlebotomist, which in turn carries a risk of exposure to blood borne infections. This risk will be minimised by a) ensuring staff are adequately trained, b) ensuring staff have been vaccinated against, and show immunity to Hepatitis B and c) having a local policy for needle stick injury which describes the process of being assessed for and receiving post exposure prophylaxis.

MRI Scans

The MRI scanner consists of a large powerful magnet. Magnetic resonance imaging uses no ionising radiation. There are, however, potential hazards associated with MRI and the scanning of participants including the presence of surgical implants, participants' clothing, jewellery (such as body piercings) bodily habitus, or medical conditions. A comprehensive list of potential risks has been compiled, and the participant will be checked against this by the operator, prior to entering the controlled areas of the MRI scanners. The MRI machine is shaped like a long tube and patients will be asked to lie in the narrow tunned in the middle. People with a history of claustrophobia may be excluded from participation in the study. All participants will still be introduced carefully to the scanner and allowed to leave at any stage, should they wish to do so. Once in the scanner, participants will be able to indicate immediately if they wish the scanning to cease by pressing a call button in their hands. All patients' cardiac implantable devices (ICD or CRT-D devices) will be set in an MRI safe mode for the duration of the scan, in accordance with the individual manufacturer's conditions and following local clinical protocols.

The use of gadolinium contrast is considered safe but there are some potential risks associated with this(68). Mild self-limiting side effects my include nausea, dizziness, headaches and very rarely (less than 1/1000) allergic reactions(69). The vast majority of gadolinium is rapidly eliminated from the body by the kidneys, but a small amount of gadolinium may remain in the brain, bones and skin for a long time (months to years). The long-term effects of this are unknown but no evidence of harm form this exists to date(70). In patients with severely impaired kidney function there is a small risk of nephrogenic systemic fibrosis but such patients are not included in this study.

CT scan

© Copyright: The University of Oxford and Oxford University Hospitals NHS Foundation Trust 2019

A computed tomography (CT) simulation scan, necessary for the treatment planning and dose calculation, will be performed with iodine contrast and delayed enhancement. Radiation is used as part of CT scanning and MR-guided radiotherapy to the stellate ganglia.

The estimated radiation dose to the patient from the CT scan is approximately 8 mSv. The risk of fatal cancer from this procedure is approximately 1 in 10,000 and equivalent to approximately 4 years' worth of background radiation (average of 2.5 mSv per year) received in the UK.

The use of iodine contrast is routine in clinical practice and safe, but there are some associated risks including allergic reactions to contrast, and contrast induced nephropathy. CT scans will be performed at sites staffed with ALS (Advanced Life Support) trained personal and equipped with appropriate resuscitations equipment, so that patients can be treated appropriately in the rare event of a severe contrast related allergic reaction. Patients at high risk of contrast induced nephropathy will be excluded from this study as per our pre-defined exclusion criteria (section 6.3)

Patient reported outcome measures

As part of this study, we will be using two PROM questionnaires.

The Kansas City Cardiomyopathy (KCCQ) questionnaire has been extensively validated to assess health status and quality of life in patients with a range of cardiomyopathies(71-74). It consists of 23 questions and has a has a recall period of 2 weeks over which patients describe the frequency and severity of their symptoms, their physical and social limitations, and how they perceive their symptoms to affect their quality of life. These domains can be combined into an Overall Summary score that combines the Total Symptom, Physical and Social Limitation, and Quality of Life scales to provide a more holistic summary of the patients' health status.

A second questionnaire will be used to screen for possible side effects following MR-guided radiotherapy to the stellate ganglia. We have designed this questionnaire by compiling symptoms that are commonly reported in trials of surgical sympathetic denervation(42, 43), and patients receiving treatment for head and neck cancers(75)

Biomarker Measurements:

The project will initially focus on plasma NPY levels, which we have significant experience of measuring. However, as other targets are identified from on-going studies then further biomarker measurements may

© Copyright: The University of Oxford and Oxford University Hospitals NHS Foundation Trust 2019

be made as required. Blood samples will be taken from a peripheral vein. Blood samples will be transported to the lab in secure containers on dry ice by a member of the study team. Here they will be centrifuged and immediately frozen to -80 °C. NPY levels will be measured using ELISA (EZHNPY-25K, Millipore) as described previously (31, 32, 76, 77).

9.5. Baseline Assessments

Visit 1 – screening visit and screening 1.5 T MRI

Eligibility of participants will be re-checked by pre-defined inclusion/exclusion criteria (see 6.2 and 6.3 for details) by the study team and MRI safety checks will be conducted. Written informed screening consent will be obtained by GCP trained investigators or research nurses who have also been trained to obtain consent for the study and procedures and listed on the study delegation log (20 minutes).

Anthropometric measurements such as height, weight, BMI will be recorded, and routine cardiovascular examination will afford the detection of any previously undiagnosed pathology that may have implications for safety. Participants will be asked to complete the Kansas City Cardiomyopathy Questionnaire (KCCQ) so assess baseline quality of life scores. Standard 12 lead electrocardiogram will be taken, which is a highly routine test, and baseline blood tests will be taken (approximately 16ml) (10 minutes)

A 1.5 T Cardiac Magnetic Resonance (CMR) scan will be performed on a 1.5 Tesla MR scanner at the OCMR with sequences optimised to minimise device related artefact to allow us to clearly visualise the stellate ganglia and heart. Participants will lie in a supine position and a dedicated coil will be placed around their chest, neck and head. Oxford University Hospitals NHS Foundation Trust has a standard operating procedure in place (developed with the Chief Investigator) for MR imaging of patients with implantable cardiac devices and this will be adhered to. The expected magnetic resonance imaging time is less than 60 -90 minutes. There is a scheduled 5–10-minute break during scanning.

We anticipate a 20-30% screening failure so we will not proceed with formal study consent until eligibility is confirmed following screening assessment. Patients who pass screening will be invited to formally enrol into the study and will proceed as outlined below. Patients who fail screening will exit at this point.

© Copyright: The University of Oxford and Oxford University Hospitals NHS Foundation Trust 2019

9.6. Subsequent Visits

Visit 2 – Radiotherapy team visit and study consent

Once Participants have been screened, they will be invited to formally enrol into the study. This process with begin with an introductory visit with the radiotherapy team. At this point patients will receive additional counselling about the radiotherapy process and possible side effects. This visit will last approximately 1 hour. If patients are still happy to proceed with the study, then written informed consent will be obtained at this point.

Visit 3 – Planning scans

A planning contrast computed tomography (CT) scan of the stellate ganglia in the neck, necessary for the treatment planning and radiotherapy dose calculation, will be performed. Patients will lie in a supine position. To ensure they are accurately positioned for the planning scan and subsequent treatment, they will have a plastic mask made to fit over their neck and shoulders to wear for this scan. (30 minutes)

For MR based radiotherapy, participants will also undergo an additional 0.35T MRI scan performed on the MR-Linac . To ensure they are accurately positioned for the planning scan and subsequent treatment, they will again require the same plastic mask to wear for this scan. Participants will lie in a supine position and a dedicated coil will be placed around their chest, neck and head. Standard operating procedure for MR imaging of patients with implantable cardiac devices will be adhered to. The expected MR imaging time is approximately 60

Visits 4 to 6 – Hypofractionated radiotherapy to the stellate ganglia

Over the course of one-week, patients will undergo up to 3 fractions of hypofractionated radiotherapy targeted bilaterally to the lower half of the stellate ganglia and T1-2 paravertebral chain (60 minutes) .

Prior to each fraction of radiotherapy blood samples (approximately 16 ml) will be taken to monitor plasma levels of NPY and other biomarkers. Routine blood tests (including renal function, full blood count and thyroid function) will also be checked prior to the first fraction of treatment. Blood samples will be labelled with the participants' study number and stored for analysis. All stored samples will be de-identified and will only be identified by a study specific participant number and will be destroyed after processing, unless participants have specifically consented to have their samples stored for use in future studies (10 minutes)

Before and after each fraction of radiotherapy a cardiovascular examination and neurological examination will be performed by one of the study doctors paying attention to signs of Horner's syndrome

© Copyright: The University of Oxford and Oxford University Hospitals NHS Foundation Trust 2019

(ptosis and pupil constriction) or upper limb sensory impairment. Cardiovascular observations including postural blood pressures and heart rate will be recorded, and an electrocardiogram will be performed. New postural hypotension defined as a symptomatic postural blood pressure drop of 20mmHg will be considered significant. (10 minutes each) If any serious treatment related adverse events are detected, then no further fractions of radiation will be delivered to that patient.

Visits 7 to 9 – safety visits

Over the course of 6 months post completion of hypofractionated radiotherapy, patients will be followed up to monitor for treatment related adverse events and to monitor biomarker response. This will comprise 3 further study visits at 6 weeks, 3 months and 6 months (or earlier if clinically indicated) following completion of radiotherapy.

During each study visit, blood samples will be taken (approximately 16 ml) to monitor plasma levels of NPY and other biomarkers. At the final 6-month study visit (or earlier if clinically indicated) routine blood tests will also be repeated (as detailed above).

Participants will be interviewed to assess for side effects from their radiotherapy and cardiac sympathetic denervation. Physical examinations and observations will be performed, and an electrocardiogram will be taken (as detailed above). Participants will also be asked to complete the KCCQ and a symptom questionnaire to screen for any side effects from their radiotherapy or cardiac sympathetic denervation.

At the 6 months visit a final 1.5T CMR scan will also be performed at OCMR to reassess cardiac function and stellate ganglia anatomy (as detailed above).

ICD interrogation and monitoring

Throughout the study duration, all patients ICDs will be monitored remotely through the pacing clinic as part of standard clinical care for these patients. Prior to patient safety visits the study team will access these records to identify any device treatments (shocks or ATP) delivered for ventricular arrhythmias and determine heart rate parameters.

© Copyright: The University of Oxford and Oxford University Hospitals NHS Foundation Trust 2019

Figure 3. Study flow chart

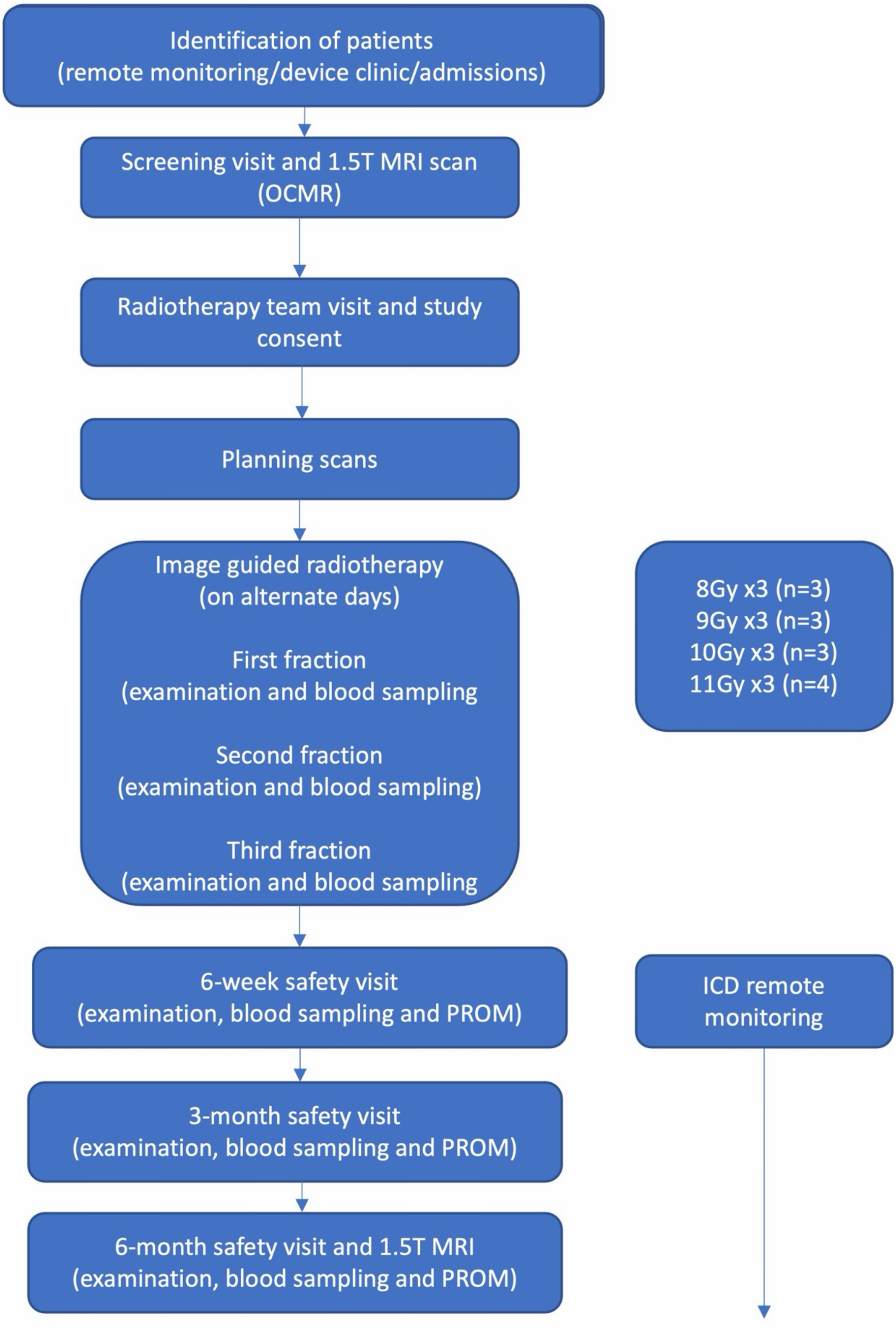

© Copyright: The University of Oxford and Oxford University Hospitals NHS Foundation Trust 2019

9.7. Sample Handling

A total of 6 samples (total volume of no more than 100 ml) will be collected during the entire study duration. Blood samples will be stored in the research facility in OCMR until processed. All stored samples for this study will only be identified by a study specific participant number and will be destroyed after processing, unless participant has given consent for use of samples in future studies when signing the consent form. All processing of these blood samples will be performed in Oxford University Laboratories, either at the John Radcliffe Hospital, or the Department of Physiology Anatomy and Genetics and will be performed by the University Research Team. Blood samples will be transported to the lab in secure containers on dry ice by a member of the study team. Here they will be centrifuged and immediately frozen to -80 °C. NPY levels will be measured using ELISA (EZHNPY-25K, Millipore) as described previously (31, 32, 76, 77).

9.8. Early Discontinuation/Withdrawal of Participants

During the course of the study a participant may choose to withdraw early from the study treatment at any time. This may happen for several reasons, including but not limited to:

- The occurrence of what the participant perceives as an intolerable AE.
- Inability to comply with study procedures
- Participant decision

Participants may choose to stop treatment and/or study assessments but may remain on study follow-up.

Participants may also withdraw their consent, meaning that they wish to withdraw from the study completely.

According to the design of the study, participants may have the following three options for withdrawal;

1) Participants may withdraw from active follow-up and further communication but allow the study team to continue to access their medical records and any relevant hospital data that is recorded as part of routine standard of care; i.e., Remote monitoring of their ICD.

2) Participants can withdraw from the study but permit data and samples obtained up until the point of withdrawal to be retained for use in the study analysis. No further data or samples would be collected after withdrawal.

3) Participants can withdraw completely from the study and withdraw the data and samples collected up until the point of withdrawal. The data and samples already collected would not be used in the final study analysis. A limitation to this withdrawal would be any data that has already been used as part of interim analysis, including by the independent safety committee.

In addition, the Investigator may discontinue a participant from the study treatment at any time if the Investigator considers it necessary for any reason including, but not limited to:

© Copyright: The University of Oxford and Oxford University Hospitals NHS Foundation Trust 2019

- Pregnancy
- Ineligibility (either arising during the study or retrospectively having been overlooked at screening)
- Significant protocol deviation
- Significant non-compliance with treatment regimen or study requirements
- Clinical decision

The type of withdrawal and reason for withdrawal will be recorded in the CRF.

Withdrawn participants will be replaced

Patients who have withdrawn from the study who also opt out of further study follow up will continue to be followed indefinitely up as part of their routine clinical care. This will include follow up under the device clinic, and where applicable by the heart failure and arrhythmia service.

9.9. Definition of End of Study

The end of study is once all patient visits are complete and all samples have been analysed.

10. SAFETY REPORTING

This study is primarily a safety study and is specifically powered to detect SAEs (see section 11.3). The safety reporting window will be from the first fraction of radiotherapy until completion of the 6 months study follow up. A 6 months SAE reporting window is felt to be sufficient as adverse events related to cardiac sympathetic denervation should be apparent during treatment or within days, weeks or months of completion of radiotherapy if we consider trigeminal neuralgia as a parallel (78). Indeed, over the course of 6 month we will monitor any adverse events and side-effects and the expectation is that side effects will improve rather than worsen over this time period, as is observed following surgical removal of the stellate ganglia(20)

With respect to adverse events following radiotherapy, we expect to detect any dose limiting toxicity within 6 weeks of completion of radiotherapy, which is an accepted monitoring period for dose escalation trials involving radiotherapy (79). In terms of stochastic adverse effects, in the long term, ionising radiation can also cause cancer which manifests itself after many years or decades and is an accepted side-effect of the treatment. It is not likely that we would detect such adverse events even if our reporting window was extended beyond 6 months. Indeed, our study population of patients with severe heart failure and recurrent ventricular arrhythmias has a particularly poor prognosis, even despite all current available medical therapies, with mortally rates observed in excess of 50% at 5 years(67). As such late stochastic events might never become apparent.

10.1. Independent safety monitoring committee

© Copyright: The University of Oxford and Oxford University Hospitals NHS Foundation Trust 2019

This study will include an independent safety monitoring committee which will consist of consultant cardiologist specialising in cardiac devices and electrophysiology [redacted] and consultant clinical oncologist specialising in the radiotherapy treatment of head and neck cancers, [redacted]. This committee will routinely meet after the completion of each dose of the dose escalation protocol (see figure 2.), and dose escalation will only occur once the committee is satisfied with safety. In addition, if any SAE (as defined in section 10.2) is detected during study follow up visits, this will trigger a safety committee review. No further patients will be recruited, and no further participants will receive radiotherapy pending outcome of this review.

10.2. Definition of Serious Adverse Events

A serious adverse event is any untoward medical occurrence that

- results in death
- is life-threatening
- requires inpatient hospitalisation or prolongation of existing hospitalisation
- results in persistent or significant disability/incapacity
- consists of a congenital anomaly or birth defect.

Other ‘important medical events’ may also be considered a serious adverse event when, based upon appropriate medical judgement, the event may jeopardise the participant and may require medical or surgical intervention to prevent one of the outcomes listed above.

NOTE: The term "life-threatening" in the definition of "serious" refers to an event in which the participant was at risk of death at the time of the event; it does not refer to an event which hypothetically might have caused death if it were more severe.

10.3. Reporting Procedures for Serious Adverse Events

A serious adverse event (SAE) occurring to a participant will be reported to the REC that gave a favourable opinion of the study where in the opinion of the Chief Investigator the event was ‘related’ (resulted from administration of any of the research procedures) and ‘unexpected’ in relation to those procedures. Reports of related and unexpected SAEs will be submitted within 15 working days of the Chief Investigator becoming aware of the event, using the HRA report of serious adverse event form (see HRA website).

10.4. Reporting Procedures for Device Related Adverse Events

All patients in this study will have MRI conditional implantable cardiac devices as defined by the inclusion criteria. All patients’ cardiac implantable devices (ICD or CRT-D devices) will be set in an MRI safe mode for the duration of the scan, in accordance with the individual manufacturer’s conditions and following local clinical protocols. Furthermore, as part of the individual planning of each participant’s radiotherapy, the radiation dose to their device will be considered and must be below their tolerance constraints as defined by the UK Consensus (63) and American Association of Physicists in Medicine (AAPM) guidelines (64).

However, it is still theoretically possible that a device complication could occur despite adherence to these protocols. If a device related complication occurs, we will notify the device manufacturer within 1

© Copyright: The University of Oxford and Oxford University Hospitals NHS Foundation Trust 2019

working day for further investigation. If the device related complications affect clinical safety of the participant, immediate appropriate clinical action will be taken, which could include admission to the cardiology ward for further assessment and treatment. If such complications amount to an SAE (as defined in 10.2) and investigators consider such complications ‘related’ (resulted from administration of any of the research procedures) and ‘unexpected’ in relation to study procedures these will also be reported to REC as outlined in section 10.3.

10.5. Follow-up of Serious Adverse Events

In the event of an SAE, participants will remain under study follow up for the duration of the study to monitor the progress of the SAE. If deemed clinically appropriate by the investigators, and with the consent of the patient, onward clinical referral to the appropriate clinical specialty will be arranged for further investigation and treatment of symptoms. This study will monitor and report on SAE which occur within the pre-specified monitoring window period as discussed in section 10.0. Following completion of the study, all patients will return to routine clinical follow up as part of their routine clinical care. This will include follow up under the device clinic and, where applicable, by the heart failure and arrhythmia service.

11. STATISTICS AND ANALYSIS

11.1. Statistical Analysis Plan (SAP)

The plan for the statistical analysis of the study are outlined below. There is not a separate SAP document in use for the trial.

11.2. Description of the Statistical Methods

To measure our primary safety endpoint, we will measure the frequency of adverse events in the 6 months post radiotherapy. Our trial is powered to detect adverse event rates of 34% (see below)

For secondary outcome measures such as changes in circulating biomarker levels (NPY) and changes in number of device therapies following radiotherapy we will use non-parametric tests such as a Wilcoxon signed rank test to determine if changes are statistically significant.

To assess correlation between changes in biomarker levels and number of device therapies we will use a correlation matrix using non-parametric correlation tests such as Spearman rank-order correlation.

11.3. Sample Size Determination

Considering that all patients will have failed pharmacological treatments to prevent VT, the population is expected to be at particularly high risk and SAE rates_≤34% (as reported for surgical CSD), and efficacy rates of 50% would be clinically acceptable. To be 80% powered to demonstrate a SAE rate not

© Copyright: The University of Oxford and Oxford University Hospitals NHS Foundation Trust 2019

exceeding 34% (range 9-34%) on a one sided, one sample test for proportions (alpha 0.05) would require 13 patients. Considering that all patients will have failed pharmacological treatments to prevent dangerous heart rhythms, the population is expected to be at particularly high risk of further dangerous heart rhythms. Efficacy rates of 50% would be clinically acceptable. We anticipate screening at least 16 patients to allow for a 20% drop out rate, **which has been costed in our BHF grant.**

11.4. Analysis populations

All participants who completed their course of radiotherapy bilaterally to the stellate ganglia (an adverse event analysis).

11.5. Decision points

Interim analysis will be performed after the completion of each dose of the dose escalation protocol. This will include assessment of safety by an independent safety committee. Study investigators will respect any recommendations from the safety committee, including a recommendation to stop the trial is there is sufficient safety concern.

11.6. Stopping rules

An early stopping rule will be set to halt protocol enrolment if ≥4 of the first 8 participants developed a related and unexpected SAE.

11.7. The Level of Statistical Significance

We are 80% powered to demonstrate a SAE rate not exceeding 34% (range 9-34%) on a one sided, one sample test for proportions (alpha 0.05).

12. DATA MANAGEMENT

The plan for the data management of the study are outlined below. There is not a separate Data Management document in use for the study. Participant data will be treated the same way irrespective of the outcome of the initial screening assessment.

12.1. Source Data

Source documents are where data are first recorded, and from which participants' CRF data are obtained. These include, but are not limited to, hospital records (from which medical history and previous and concurrent medication may be summarised into the CRF), clinical and office charts, laboratory and pharmacy records, diaries, microfiches, radiographs, and correspondence.

CRF entries will be considered source data if the CRF is the site of the original recording (e.g. there is no other written or electronic record of data). All documents will be stored safely in confidential conditions.

© Copyright: The University of Oxford and Oxford University Hospitals NHS Foundation Trust 2019

On all study-specific documents, other than the signed consent, the participant will be referred to by the study participant number/code, not by name.

12.2. Access to Data

Direct access will be granted to authorised representatives from the Sponsor and host institution for monitoring and/or audit of the study to ensure compliance with regulations.

12.3. Data Recording and Record Keeping

All study data will be initially collected and entered onto a password protected Excel document and saved on a university computer that requires a user log-in.

Personal data will be recorded electronically on data forms that are entered into an access-restricted computer and encrypted. The participants will be identified by a study specific participant number in any database.

During the study, data containing personal information such as name of the participant and contact details will be stored securely with restricted user access. Personal information will be removed from study documents such as source documents as soon as practical, and then replaced with an ID number from a linkage document kept separately and securely. Documentation containing such personal information will be destroyed or de-identified 6-12 months after the end of the study.

Documents containing personal or identifying data such as the code break and consent forms will also be stored in cabinets with restricted user access during the study.

At the end of ethical approval, the study files will be archived in the Div. of Cardiovascular Medicine, University of Oxford for five years, after which time the custodian will agree a date for destruction and data will be destroyed confidentially.

Electronic data will be held on secure network drives/hard disks/ servers on password protected computers within locked offices. Backup copies of files will be made regularly weekly and stored on a different server/external hard drive. These back up locations will be subject to the same security principles as the primary locations. When datasets are complete, the primary copy will remain at the study site where it will be transferred onto optical media, e.g. DVD/external drive and undergo archiving for hard copy data. Any copies leaving the study site will be completely anonymised/de-identified.

Where participants have consented for storage and use of blood samples for future research, and further analysis beyond the end of the study as potential novel biomarkers are identified. The consent form will be retained for the life of the sample to meet HTA traceability requirements.

© Copyright: The University of Oxford and Oxford University Hospitals NHS Foundation Trust 2019

13. QUALITY ASSURANCE PROCEDURES

The study may be monitored, or audited in accordance with the current approved protocol, GCP, relevant regulations and standard operating procedures. Data collected for the study may be reviewed for auditing and monitoring by authorised persons from the sponsor, regulatory authorities or their local host institutions to make sure that it is being carried out correctly. All investigators have a duty of confidentiality to research participants and nothing that could reveal their identity would be disclosed outside the research study team or site.

In addition, depending on the complexity, scale and risks inherent to the research study the quality assurance procedures may include a risk adaptive approach based on a formal risk assessment, planned monitoring activities (onsite and / or central monitoring) and the involvement of a number of study oversight committees.

13.1. Risk assessment

A risk assessment and monitoring plan will be prepared before the study opens and will be reviewed as necessary over the course of the study to reflect significant changes to the protocol or outcomes of monitoring activities.

13.2. Study monitoring

Regular monitoring will be performed according to the study specific Monitoring Plan. Data will be evaluated for compliance with the protocol and accuracy in relation to source documents. Following written standard operating procedures, the monitors will verify that the clinical study is conducted and data are generated, documented and reported in compliance with the protocol, GCP and the applicable regulatory requirements.

13.3. Study Committees

This study will include an independent safety monitoring committee which will consist of consultant cardiologist specialising in complex implantable cardiac devices and electrophysiology [redacted], and a consultant clinical oncologist specialising in the radiotherapy treatment of head and neck cancers [redacted]. This committee will routinely meet after the completion of each dose of the dose escalation protocol (see figure 2.), and dose escalation will only occur once the committee is satisfied with safety. In addition, if any SAE (as defined in section 10.2) is detected during study follow up visits, this will trigger a safety committee review. No further patients will be recruited and no further patients will receive radiotherapy pending outcome of this review.

14. PROTOCOL DEVIATIONS

© Copyright: The University of Oxford and Oxford University Hospitals NHS Foundation Trust 2019

A study related deviation is a departure from the ethically approved study protocol or other study document or process (e.g. consent process or administration of study intervention) or from Good Clinical Practice (GCP) or any applicable regulatory requirements. Any deviations from the protocol will be documented in a protocol deviation form and filed in the study master file. This will be done in line with the Oxford university standard operating procedure for Serious Breach of Good Clinical Practice or the Trial Protocol (Core SOP 008 Version 4.0)

15. SERIOUS BREACHES

A "serious breach" is a breach of the protocol or of the conditions or principles of Good Clinical Practice which is likely to affect to a significant degree –

(a) the safety or physical or mental integrity of the trial subjects; or

(b) the scientific value of the research.

In the event that a serious breach is suspected the Sponsor must be contacted within 1 working day. In collaboration with the C.I., the serious breach will be reviewed by the Sponsor and, if appropriate, the Sponsor will report it to the approving REC committee and the relevant NHS host organisation within seven calendar days.

16. ETHICAL AND REGULATORY CONSIDERATIONS

16.1. Declaration of Helsinki

The Investigator will ensure that this study is conducted in accordance with the principles of the Declaration of Helsinki.

16.2. Guidelines for Good Clinical Practice

The Investigator will ensure that this study is conducted in accordance with relevant regulations and with Good Clinical Practice.

16.3. Approvals

Following Sponsor approval, the protocol, informed consent form, participant information sheet will be submitted to an appropriate Research Ethics Committee (REC) and host institutions for written approval.

The Investigator will submit and, where necessary, obtain approval from the above parties for all substantial amendments to the original approved documents.

16.4. Other Ethical Considerations

16.4.1. Cardiac sympathetic denervation

The aim of delivering radiotherapy to the stellate ganglion in this study is to achieve functional sympathetic denervation of the heart. Reports about the type and frequency of complications arising from cardiac sympathetic denervation come from case series of surgical removal of the stellate ganglion.

© Copyright: The University of Oxford and Oxford University Hospitals NHS Foundation Trust 2019

A large number of complications associated this with are due to surgical complications such as pneumothorax, pleural effusion and infection. These do not apply to our study, and indeed are part of the motivation for this study to achieve CSD in a non-invasive manner.

The primary non-surgical complications associated with CSD are off site neurological effects and hypotension. A recent systematic review including 173 patients who had surgical CSD estimated the risk of hypotension as 9%, and off-site neurological effects as follows: Horner's syndrome 3%, and upper limb and face hyperalgesia 4% and hyperhidrosis 3%(19). No procedural deaths were reported. The risk of Horner's syndrome can be mitigated by sparing the cranial portion of the stellate ganglion and sparing the T3-T4 paravertebral ganglia may reduce off site neurological complications while still achieving sufficient CSD(30).

Importantly most significant neurological and haemodynamic complications of CSD are transient in nature. In a case series of 121 patients who underwent CSD, 5 patients developed Horner's syndrome, but this completely resolved in 4 of these patients by 6 months (20). It seems that residual cardiac sympathetic innervation to the heart from the middle cervical ganglia (which are preserved following CSD) is sufficient to maintain haemodynamic stability(80). Finally, and importantly from a patient's perspective, most patients who undergo CSD are satisfied with the procedure, with no negative impact on quality of life even in the presence of some persistent side-effects (42). Interestingly this compares favourably to findings of worsened QOL scores associated with ICD implantation which is currently a widely accepted treatment for patients at risk of ventricular arrhythmias(81, 82). Indeed, the use of VATS sympathetic cardiac denervation already forms part of guideline endorsed treatment of Long QT syndrome and catecholaminergic polymorphic VT, with a similar level of recommendation as ICD implantation. (83)

Our study is specifically designed to minimise the risk of these complications. We will only target the T1-2 gangila of paravertebral chain and aim to spare the cranial portion of the stellate ganglion. However the stellate ganglion is shaped differently in different individuals with variable degrees of fusion between the lower cervical and thoracic ganglia(84). The level of sparing will thus vary from individual to individual based on their anatomy. By achieving CSD in a staged and stepwise manner over 3 fractions of radiotherapy we aim to detect any complications early before permanent CSD is achieved, thus mitigating the severity of these complications. Finally, the radiotherapy doses we have chosen for this study are at the lower tolerance limit for neuronal tissue, and lower than does used in previous studies (56) as we aim for stellate ganglion modification rather than complete destruction. Indeed, it is our hypothesis that Image-guided hypofractionated radiotherapy for stellate ganglion modification will result in a lower complication rate than that so far observed following surgical CSD, and one of the co-primary outcomes of this study is treatment related adverse effects.

16.4.2. Incidental findings

It is possible that the study investigations could uncover unexpected disease findings on the MRI scan, blood tests or other research procedures. OCMR has an established SOP to be followed in the instance of an unexpected abnormality found on a research scan. If any evidence of an unexpected abnormality is detected on the MRI scans the investigators will not attempt to interpret them. They will contact the site MR technologist, consultant, or radiologist for conformation of an abnormality prior to disclosing any

© Copyright: The University of Oxford and Oxford University Hospitals NHS Foundation Trust 2019

problem to the participant. For any abnormalities confirmed, a designated clinical specialist will discuss the implications with the participant and may arrange for further investigations as necessary. Participants will be aware from the PIL that research scans and procedures are not for diagnostic purposes, and therefore are not a substitute for a clinical appointment. Investigators will gain permission from the participant to contact their general practitioner (GP) directly so that the GP can then arrange appropriate management.

16.5. Reporting

The CI shall submit once a year throughout the study, or on request, an Annual Progress report to the REC Committee, HRA (where required) host organisation, Sponsor and funder (where required). In addition, an End of Study notification and final report will be submitted to the same parties.

16.6. Transparency in Research

Prior to the recruitment of the first participant, the trial will have been registered on a publicly accessible database.

16.7. Participant Confidentiality

The study will comply with the UK General Data Protection Regulation (GDPR) and Data Protection Act 2018, which require data to be de-identified as soon as it is practical to do so. The processing of the personal data of participants will be minimised by making use of a unique participant study number only on all study documents and any electronic database(s), (with the exception of the CRF, where participant initials may be added). All documents will be stored securely and only accessible by study staff and authorised personnel. The study staff will safeguard the-privacy of participants' personal data.

Responsible members of the University of Oxford (the sponsor) may be given access to data for monitoring and/or audit of the study to ensure we are complying with regulations and good practice.

Blood samples will be de-identified and will only be identified by a study specific participant number.

16.8. Expenses and Benefits

Reasonable travel expenses for any visits additional to normal care will be reimbursed on production of receipts, or a mileage allowance provided as appropriate up to a maximum of £30.00 per visit.

17. FINANCE AND INSURANCE

17.1. Funding

© Copyright: The University of Oxford and Oxford University Hospitals NHS Foundation Trust 2019

This research is funded in full by the British Heart Foundation as part of a BHF Clinical Research Training Fellowship (FS/CRTF/22/24437)

17.2. Insurance

The University has a specialist insurance policy in place which would operate in the event of any participant suffering harm as a result of their involvement in the research (Newline Underwriting Management Ltd, at Lloyd's of London). NHS indemnity operates in respect of the clinical treatment that is provided.

17.3. Contractual arrangements

Appropriate contractual arrangements will be put in place with all third parties.

18. PUBLICATION POLICY

The Investigators will be involved in reviewing drafts of the manuscripts, abstracts, press releases and any other publications arising from the study. Authors will acknowledge that the study was funded by the British Heart Foundation. Authorship will be determined in accordance with the ICMJE guidelines and other contributors will be acknowledged.

19. DEVELOPMENT OF A NEW PRODUCT/ PROCESS OR THE GENERATION OF INTELLECTUAL PROPERTY

Ownership of IP generated by employees of the University vests in the University. The University will ensure appropriate arrangements are in place as regards any new IP arising from the trial.

19. ARCHIVING

At the end of ethical approval, the study files will be archived in the Div. of Cardiovascular Medicine, University of Oxford for five years, after which time the custodian will agree a date for destruction and data will be destroyed confidentially.

Electronic data will be held on secure network drives/hard disks/ servers on password protected computers within locked offices. Backup copies of files will be made regularly weekly and stored on a different server/external hard drive. These back up locations will be subject to the same security principles as the primary locations. When datasets are complete, the primary copy will remain at the study site where it will be transferred onto optical media, e.g. DVD/external drive and undergo archiving for hard copy data. Any copies leaving the study site will be completely anonymised/de-identified.

20. REFERENCES

© Copyright: The University of Oxford and Oxford University Hospitals NHS Foundation Trust 2019

1. Adabag AS, Luepker RV, Roger VL, Gersh BJ. Sudden cardiac death: epidemiology and risk factors. Nat Rev Cardiol. 2010;7(4):216-25.
2. Paratz ED, Rowsell L, Zentner D, Parsons S, Morgan N, Thompson T, et al. Cardiac arrest and sudden cardiac death registries: a systematic review of global coverage. Open Heart. 2020;7(1):e001195.
3. Excellence NIoHaC. Chronic heart failure in adults: diagnosis and management (NG106). wwwniceorguk/guidance/ng106. 2018.
4. BHF. British Heart Foundation UK Factsheet March 2021. https://wwwbhforguk/what-we-do/our-research/heart-statistics. 2021.
5. Effect of metoprolol CR/XL in chronic heart failure: Metoprolol CR/XL Randomised Intervention Trial in-Congestive Heart Failure (MERIT-HF). The Lancet. 1999;353(9169):2001-7.
6. The Cardiac Insufficiency Bisoprolol Study II (CIBIS-II): a randomised trial. The Lancet. 1999;353(9146):9-13.
7. Mason JW. A comparison of seven antiarrhythmic drugs in patients with ventricular tachyarrhythmias. Electrophysiologic Study versus Electrocardiographic Monitoring Investigators. N Engl J Med. 1993;329(7):452-8.
8. Singh SN, Fletcher RD, Fisher SG, Singh BN, Lewis HD, Deedwania PC, et al. Amiodarone in patients with congestive heart failure and asymptomatic ventricular arrhythmia. Survival Trial of Antiarrhythmic Therapy in Congestive Heart Failure. N Engl J Med. 1995;333(2):77-82.
9. Bardy GH, Lee KL, Mark DB, Poole JE, Packer DL, Boineau R, et al. Amiodarone or an implantable cardioverter-defibrillator for congestive heart failure. N Engl J Med. 2005;352(3):225-37.
10. Echt DS, Liebson PR, Mitchell LB, Peters RW, Obias-Manno D, Barker AH, et al. Mortality and morbidity in patients receiving encainide, flecainide, or placebo. The Cardiac Arrhythmia Suppression Trial. N Engl J Med. 1991;324(12):781-8.
11. Moss AJ, Zareba W, Hall WJ, Klein H, Wilber DJ, Cannom DS, et al. Prophylactic implantation of a defibrillator in patients with myocardial infarction and reduced ejection fraction. N Engl J Med. 2002;346(12):877-83.
12. Poole JE, Johnson GW, Hellkamp AS, Anderson J, Callans DJ, Raitt MH, et al. Prognostic importance of defibrillator shocks in patients with heart failure. N Engl J Med. 2008;359(10):1009-17.
13. Connolly SJ, Dorian P, Roberts RS, Gent M, Bailin S, Fain ES, et al. Comparison of beta-blockers, amiodarone plus beta-blockers, or sotalol for prevention of shocks from implantable cardioverter defibrillators: the OPTIC Study: a randomized trial. JAMA. 2006;295(2):165-71.
14. Shivkumar K. Catheter Ablation of Ventricular Arrhythmias. N Engl J Med. 2019;380(16):1555-64.
15. Kuck KH, Schaumann A, Eckardt L, Willems S, Ventura R, Delacretaz E, et al. Catheter ablation of stable ventricular tachycardia before defibrillator implantation in patients with coronary heart disease (VTACH): a multicentre randomised controlled trial. Lancet. 2010;375(9708):31-40.
16. Kuck KH, Tilz RR, Deneke T, Hoffmann BA, Ventura R, Hansen PS, et al. Impact of Substrate Modification by Catheter Ablation on Implantable Cardioverter-Defibrillator Interventions in Patients With Unstable Ventricular Arrhythmias and Coronary Artery Disease: Results From the Multicenter Randomized Controlled SMS (Substrate Modification Study). Circ Arrhythm Electrophysiol. 2017;10(3).
17. Reddy VY, Reynolds MR, Neuzil P, Richardson AW, Taborsky M, Jongnarangsin K, et al. Prophylactic catheter ablation for the prevention of defibrillator therapy. N Engl J Med. 2007;357(26):2657-65.
18. Sapp JL, Wells GA, Parkash R, Stevenson WG, Blier L, Sarrazin JF, et al. Ventricular Tachycardia Ablation versus Escalation of Antiarrhythmic Drugs. N Engl J Med. 2016;375(2):111-21.
19. Shah R, Assis F, Alugubelli N, Okada DR, Cardoso R, Shivkumar K, et al. Cardiac sympathetic denervation for refractory ventricular arrhythmias in patients with structural heart disease: A systematic review. Heart Rhythm. 2019;16(10):1499-505.
20. Vaseghi M, Barwad P, Malavassi Corrales FJ, Tandri H, Mathuria N, Shah R, et al. Cardiac Sympathetic Denervation for Refractory Ventricular Arrhythmias. J Am Coll Cardiol. 2017;69(25):3070-80.
21. Herring N, Kalla M, Paterson DJ. The autonomic nervous system and cardiac arrhythmias: current concepts and emerging therapies. Nat Rev Cardiol. 2019;16(12):707-26.

© Copyright: The University of Oxford and Oxford University Hospitals NHS Foundation Trust 2019

22. Shiferaw Y, Aistrup GL, Wasserstrom JA. Intracellular Ca2+ waves, afterdepolarizations, and triggered arrhythmias. Cardiovasc Res. 2012;95(3):265-8.
23. Tsien RW, Bean BP, Hess P, Lansman JB, Nilius B, Nowycky MC. Mechanisms of calcium channel modulation by beta-adrenergic agents and dihydropyridine calcium agonists. J Mol Cell Cardiol. 1986;18(7):691-710.
24. Lubbe WF, Podzuweit T, Opie LH. Potential arrhythmogenic role of cyclic adenosine monophosphate (AMP) and cytosolic calcium overload: implications for prophylactic effects of beta-blockers in myocardial infarction and proarrhythmic effects of phosphodiesterase inhibitors. J Am Coll Cardiol. 1992;19(7):1622-33.
25. Priori SG, Mantica M, Schwartz PJ. Delayed afterdepolarizations elicited in vivo by left stellate ganglion stimulation. Circulation. 1988;78(1):178-85.
26. Sanguinetti MC, Jurkiewicz NK, Scott A, Siegl PK. Isoproterenol antagonizes prolongation of refractory period by the class III antiarrhythmic agent E-4031 in guinea pig myocytes. Mechanism of action. Circ Res. 1991;68(1):77-84.
27. Ng GA, Mantravadi R, Walker WH, Ortin WG, Choi BR, de Groat W, et al. Sympathetic nerve stimulation produces spatial heterogeneities of action potential restitution. Heart Rhythm. 2009;6(5):696-706.
28. Yagishita D, Chui RW, Yamakawa K, Rajendran PS, Ajijola OA, Nakamura K, et al. Sympathetic nerve stimulation, not circulating norepinephrine, modulates T-peak to T-end interval by increasing global dispersion of repolarization. Circ Arrhythm Electrophysiol. 2015;8(1):174-85.
29. Coote JH, Chauhan RA. The sympathetic innervation of the heart: Important new insights. Auton Neurosci. 2016;199:17-23.
30. Buckley U, Yamakawa K, Takamiya T, Andrew Armour J, Shivkumar K, Ardell JL. Targeted stellate decentralization: Implications for sympathetic control of ventricular electrophysiology. Heart Rhythm. 2016;13(1):282-8.
31. Kalla M, Hao G, Tapoulal N, Tomek J, Liu K, Woodward L, et al. The cardiac sympathetic co-transmitter neuropeptide Y is pro-arrhythmic following ST-elevation myocardial infarction despite beta-blockade. Eur Heart J. 2020;41(23):2168-79.
32. Ajijola OA, Chatterjee NA, Gonzales MJ, Gornbein J, Liu K, Li D, et al. Coronary Sinus Neuropeptide Y Levels and Adverse Outcomes in Patients With Stable Chronic Heart Failure. JAMA Cardiol. 2020;5(3):318-25.
33. Bundgaard JS, Jacobsen PK, Grand J, Lindholm MG, Hassager C, Pehrson S, et al. Deep sedation as temporary bridge to definitive treatment of ventricular arrhythmia storm. Eur Heart J Acute Cardiovasc Care. 2020;9(6):657-64.
34. Bourke T, Vaseghi M, Michowitz Y, Sankhla V, Shah M, Swapna N, et al. Neuraxial modulation for refractory ventricular arrhythmias: value of thoracic epidural anesthesia and surgical left cardiac sympathetic denervation. Circulation. 2010;121(21):2255-62.
35. Do DH, Bradfield J, Ajijola OA, Vaseghi M, Le J, Rahman S, et al. Thoracic Epidural Anesthesia Can Be Effective for the Short-Term Management of Ventricular Tachycardia Storm. J Am Heart Assoc. 2017;6(11).
36. Vaseghi M, Gima J, Kanaan C, Ajijola OA, Marmureanu A, Mahajan A, et al. Cardiac sympathetic denervation in patients with refractory ventricular arrhythmias or electrical storm: intermediate and long-term follow-up. Heart Rhythm. 2014;11(3):360-6.
37. Schwartz PJ, Locati EH, Moss AJ, Crampton RS, Trazzi R, Ruberti U. Left cardiac sympathetic denervation in the therapy of congenital long QT syndrome. A worldwide report. Circulation. 1991;84(2):503-11.
38. Wilde AA, Bhuiyan ZA, Crotti L, Facchini M, De Ferrari GM, Paul T, et al. Left cardiac sympathetic denervation for catecholaminergic polymorphic ventricular tachycardia. N Engl J Med. 2008;358(19):2024-9.
39. Assis FR, Sharma A, Shah R, Akhtar T, Adari S, Calkins H, et al. Long-Term Outcomes of Bilateral Cardiac Sympathetic Denervation for Refractory Ventricular Tachycardia. JACC Clin Electrophysiol. 2021;7(4):463-70.

© Copyright: The University of Oxford and Oxford University Hospitals NHS Foundation Trust 2019

40. Kochav SM, Garan H, Gorenstein LA, Wan EY, Yarmohammadi H. Cardiac Sympathetic Denervation for the Management of Ventricular Arrhythmias. J Interv Card Electrophysiol. 2022.
41. Schwartz PJ, Ackerman MJ. Cardiac sympathetic denervation in the prevention of genetically mediated life-threatening ventricular arrhythmias. Eur Heart J. 2022;43(22):2096-102.
42. Antiel RM, Bos JM, Joyce DD, Owen HJ, Roskos PL, Moir C, et al. Quality of life after videoscopic left cardiac sympathetic denervation in patients with potentially life-threatening cardiac channelopathies/cardiomyopathies. Heart Rhythm. 2016;13(1):62-9.
43. Waddell-Smith KE, Ertresvaag KN, Li J, Chaudhuri K, Crawford JR, Hamill JK, et al. Physical and Psychological Consequences of Left Cardiac Sympathetic Denervation in Long-QT Syndrome and Catecholaminergic Polymorphic Ventricular Tachycardia. Circ Arrhythm Electrophysiol. 2015;8(5):1151-8.
44. Nonoguchi NM, Adachi M, Nogami A, Komatsu Y, Sato T, Ueda A, et al. Stellate Ganglion Phototherapy Using Low-Level Laser: A Novel Rescue Therapy for Patients With Refractory Ventricular Arrhythmias. JACC Clin Electrophysiol. 2021;7(10):1297-308.
45. Benedict SH, Yenice KM, Followill D, Galvin JM, Hinson W, Kavanagh B, et al. Stereotactic body radiation therapy: the report of AAPM Task Group 101. Med Phys. 2010;37(8):4078-101.
46. Franzese C, Balermpas P. Stereotactic ablative radiotherapy for treating primary head and neck cancer and locoregional recurrence: A comprehensive review of the literature. Clinical and Translational Radiation Oncology. 2024;46:100766.
47. Kavanagh BD, Timmerman RD. Stereotactic body radiation therapy: Lippincott Williams & Wilkins; 2005.
48. Indik JH, Gimbel JR, Abe H, Alkmim-Teixeira R, Birgersdotter-Green U, Clarke GD, et al. 2017 HRS expert consensus statement on magnetic resonance imaging and radiation exposure in patients with cardiovascular implantable electronic devices. Heart Rhythm. 2017;14(7):e97-e153.
49. Yang B, Yuan J, Cheung KY, Huang CY, Poon DMC, Yu SK. Magnetic Resonance-Guided Radiation Therapy of Patients With Cardiovascular Implantable Electronic Device on a 1.5 T Magnetic Resonance-Linac. Pract Radiat Oncol. 2022;12(1):e56-e61.
50. Robinson CG, Samson PP, Moore KMS, Hugo GD, Knutson N, Mutic S, et al. Phase I/II Trial of Electrophysiology-Guided Noninvasive Cardiac Radioablation for Ventricular Tachycardia. Circulation. 2019;139(3):313-21.
51. Cuculich PS, Schill MR, Kashani R, Mutic S, Lang A, Cooper D, et al. Noninvasive Cardiac Radiation for Ablation of Ventricular Tachycardia. N Engl J Med. 2017;377(24):2325-36.
52. Gach HM, Green OL, Cuculich PS, Wittland EJ, Marko A, Luchtefeld ME, et al. Lessons Learned From the First Human Low-Field MRI Guided Radiation Therapy of the Heart in the Presence of an Implantable Cardiac Defibrillator. Pract Radiat Oncol. 2019;9(4):274-9.
53. Mayinger M, Kovacs B, Tanadini-Lang S, Ehrbar S, Wilke L, Chamberlain M, et al. First magnetic resonance imaging-guided cardiac radioablation of sustained ventricular tachycardia. Radiother Oncol. 2020;152:203-7.
54. Radiologists FoCOoTRCo. Recommendations for using radiotherapy for benign disease in the UK. The Royal College of Radiologists. 2023.
55. van der Ree MH, Dieleman EMT, Visser J, Planken RN, Boekholdt SM, de Bruin-Bon RHA, et al. Non-invasive stereotactic arrhythmia radiotherapy for ventricular tachycardia: results of the prospective STARNL-1 trial. Europace. 2023;25(3):1015-24.
56. Hudec M, Jiravsky O, Spacek R, Neuwirth R, Knybel L, Sknouril L, et al. Chronic refractory angina pectoris treated by bilateral stereotactic radiosurgical stellate ganglion ablation: first-in-man case report. Eur Heart J Case Rep. 2021;5(8):ytab184.
57. Diez P, Hanna GG, Aitken KL, van As N, Carver A, Colaco RJ, et al. UK 2022 Consensus on Normal Tissue Dose-Volume Constraints for Oligometastatic, Primary Lung and Hepatocellular Carcinoma Stereotactic Ablative Radiotherapy. Clin Oncol (R Coll Radiol). 2022;34(5):288-300.
58. Maier JG, Perry RH, Saylor W, Sulak MH. Radiation myelitis of the dorsolumbar spinal cord. Radiology. 1969;93(1):153-60.

© Copyright: The University of Oxford and Oxford University Hospitals NHS Foundation Trust 2019

59. Parsons JT, Bova FJ, Fitzgerald CR, Mendenhall WM, Million RR. Radiation optic neuropathy after megavoltage external-beam irradiation: analysis of time-dose factors. Int J Radiat Oncol Biol Phys. 1994;30(4):755-63.
60. Stoll BA, Andrews JT. Radiation-induced Peripheral Neuropathy. Br Med J. 1966;1(5491):834-7.
61. Salazar OM, Rubin P, Feldstein ML, Pizzutiello R. High dose radiation therapy in the treatment of malignant gliomas: final report. Int J Radiat Oncol Biol Phys. 1979;5(10):1733-40.
62. Tuleasca C, Paddick I, Hopewell JW, Jones B, Millar WT, Hamdi H, et al. Establishment of a Therapeutic Ratio for Gamma Knife Radiosurgery of Trigeminal Neuralgia: The Critical Importance of Biologically Effective Dose Versus Physical Dose. World Neurosurg. 2020;134:e204-e13.
63. Lester J. Management of cancer patients receiving radiotherapy with a cardiac implanted electronic device- A clinical guideline. Royal College of Radiologists. 2015.
64. Miften M, Mihailidis D, Kry SF, Reft C, Esquivel C, Farr J, et al. Management of radiotherapy patients with implanted cardiac pacemakers and defibrillators: A Report of the AAPM TG-203(dagger). Med Phys. 2019;46(12):e757-e88.
65. Kodani N, Yamazaki H, Tsubokura T, Shiomi H, Kobayashi K, Nishimura T, et al. Stereotactic body radiation therapy for head and neck tumor: disease control and morbidity outcomes. J Radiat Res. 2011;52(1):24-31.
66. Kim JH, Kim MS, Yoo SY, Lim SM, Lee GH, Yi KH. Stereotactic body radiotherapy for refractory cervical lymph node recurrence of nonanaplastic thyroid cancer. Otolaryngol Head Neck Surg. 2010;142(3):338-43.
67. Saxon LA, Hayes DL, Gilliam FR, Heidenreich PA, Day J, Seth M, et al. Long-term outcome after ICD and CRT implantation and influence of remote device follow-up: the ALTITUDE survival study. Circulation. 2010;122(23):2359-67.
68. Radiologists TRCo. Guidance on gadolinium-based contrast agent administration to adult patients. 2019.
69. Behzadi AH, Zhao Y, Farooq Z, Prince MR. Immediate Allergic Reactions to Gadolinium-based Contrast Agents: A Systematic Review and Meta-Analysis. Radiology. 2018;286(2):471-82.
70. Welk B, McArthur E, Morrow SA, MacDonald P, Hayward J, Leung A, et al. Association Between Gadolinium Contrast Exposure and the Risk of Parkinsonism. JAMA. 2016;316(1):96-8.
71. Joseph SM, Novak E, Arnold SV, Jones PG, Khattak H, Platts AE, et al. Comparable performance of the Kansas City Cardiomyopathy Questionnaire in patients with heart failure with preserved and reduced ejection fraction. Circ Heart Fail. 2013;6(6):1139-46.
72. Nassif M, Fine JT, Dolan C, Reaney M, Addepalli P, Allen VD, et al. Validation of the Kansas City Cardiomyopathy Questionnaire in Symptomatic Obstructive Hypertrophic Cardiomyopathy. JACC Heart Fail. 2022;10(8):531-9.
73. Green CP, Porter CB, Bresnahan DR, Spertus JA. Development and evaluation of the Kansas City Cardiomyopathy Questionnaire: a new health status measure for heart failure. J Am Coll Cardiol. 2000;35(5):1245-55.
74. Spertus JA, Jones PG, Sandhu AT, Arnold SV. Interpreting the Kansas City Cardiomyopathy Questionnaire in Clinical Trials and Clinical Care: JACC State-of-the-Art Review. J Am Coll Cardiol. 2020;76(20):2379-90.
75. Singer S, Amdal CD, Hammerlid E, Tomaszewska IM, Castro Silva J, Mehanna H, et al. International validation of the revised European Organisation for Research and Treatment of Cancer Head and Neck Cancer Module, the EORTC QLQ-HN43: Phase IV. Head Neck. 2019;41(6):1725-37.
76. Herring N, Tapoulal N, Kalla M, Ye X, Borysova L, Lee R, et al. Neuropeptide-Y causes coronary microvascular constriction and is associated with reduced ejection fraction following ST-elevation myocardial infarction. Eur Heart J. 2019;40(24):1920-9.
77. Cuculi F, Herring N, De Caterina AR, Banning AP, Prendergast BD, Forfar JC, et al. Relationship of plasma neuropeptide Y with angiographic, electrocardiographic and coronary physiology indices of reperfusion during ST elevation myocardial infarction. Heart. 2013;99(16):1198-203.
78. Tuleasca C, Regis J, Sahgal A, De Salles A, Hayashi M, Ma L, et al. Stereotactic radiosurgery for trigeminal neuralgia: a systematic review. J Neurosurg. 2018;130(3):733-57.

© Copyright: The University of Oxford and Oxford University Hospitals NHS Foundation Trust 2019

79. Bauman GS, Corkum MT, Fakir H, Nguyen TK, Palma DA. Ablative radiation therapy to restrain everything safely treatable (ARREST): study protocol for a phase I trial treating polymetastatic cancer with stereotactic radiotherapy. BMC Cancer. 2021;21(1):405.
80. Irie T, Yamakawa K, Hamon D, Nakamura K, Shivkumar K, Vaseghi M. Cardiac sympathetic innervation via middle cervical and stellate ganglia and antiarrhythmic mechanism of bilateral stellectomy. Am J Physiol Heart Circ Physiol. 2017;312(3):H392-H405.
81. Schron EB, Exner DV, Yao Q, Jenkins LS, Steinberg JS, Cook JR, et al. Quality of life in the antiarrhythmics versus implantable defibrillators trial: impact of therapy and influence of adverse symptoms and defibrillator shocks. Circulation. 2002;105(5):589-94.
82. Namerow PB, Firth BR, Heywood GM, Windle JR, Parides MK. Quality-of-life six months after CABG surgery in patients randomized to ICD versus no ICD therapy: findings from the CABG Patch Trial. Pacing Clin Electrophysiol. 1999;22(9):1305-13.
83. Zeppenfeld K, Tfelt-Hansen J, de Riva M, Winkel BG, Behr ER, Blom NA, et al. 2022 ESC Guidelines for the management of patients with ventricular arrhythmias and the prevention of sudden cardiac death. Eur Heart J. 2022;43(40):3997-4126.
84. Hogan QH, Erickson SJ. MR imaging of the stellate ganglion: normal appearance. AJR Am J Roentgenol. 1992;158(3):655-9.

21. APPENDIX A: SCHEDULE OF STUDY PROCEDURES

**Procedures**	**Visits**								
	**Screening visit 1**	**Visit 2**	**Visit 3**	**Visit 4**	**Visit 5**	**Visit 6**	**Visit 7**	**Visit 8**	**Visit 9**
Screening consent for trial	X								
Informed consent for the trial		X							
Demographics	X								
Medical history	X								
Medication history	X								X
Physical examination	X			X	X	X	X	X	X
ECG	X			X	X	X	X	X	X
Laboratory tests	X			X	X	X	X	X	X
Eligibility assessment	X								
Planning scans			X						
1.5T MRI scan	X								x
Radiotherapy treatment				X	X	X			
Adverse event assessments				X	X	X	X	X	X
Symptom questionnaire				X	X	X	X	X	X

© Copyright: The University of Oxford and Oxford University Hospitals NHS Foundation Trust 2019

KCCQ -23	X								X

22. APPENDIX B: AMENDMENT HISTORY

Amendment No.	Protocol Version No.	Date issued	Author(s) of changes	Details of Changes made
1	2.0		BB, NH, AS	Addition of TrueBeam CT guided radiotherapy platform to the study

List details of all protocol amendments here whenever a new version of the protocol is produced. This is not necessary prior to initial REC / HRA submission.

Protocol amendments must be submitted to the Sponsor for approval prior to submission to the REC committee and HRA (where required).

© Copyright: The University of Oxford and Oxford University Hospitals NHS Foundation Trust 2019
