# Supplementary material for: Protocol of the RADIO-STAR trial: a phase 1 safety and dose finding study of hypofractionated radiotherapy to the stellate ganglia for the treatment of ventricular arrhythmia
Source: BMJ Open. 2026 Feb 25;16(2):e110958. doi: 10.1136/bmjopen-2025-110958 (PMC12959014; doi:10.1136/bmjopen-2025-110958)
Supplement: online supplemental file 3 [file bmjopen-16-2-s003.pdf]

DEPARTMENT OF PHYSIOLOGY, ANATOMY AND GENETICS
Defining Excellence
Oxford Anatomy and Physiology ranked #1 in the QS World University Rankings by subject 2017, 2018

UNIVERSITY OF OXFORD

Symptom Questionnaire

Department of Physiology, Anatomy and Genetics
Sherrington Building
Parks Road
Oxford
OX1 3PT

Participant ID ☐☐☐☐☐☐

Date: ☐☐/☐☐/☐☐☐☐

Visit number ☐☐ - Before / After

Nerve related side effects	
1. Have you noticed a change in the amount you are sweating in your hands, arms and face?	No<br>Yes (Mild 1 2 3 4 5 Severe)
2. Have you suffered from dry skin over your hands, arm or face?	No<br>Yes (Mild 1 2 3 4 5 Severe)
3. Have you noticed a change in the colour in your face?	No<br>Yes (Mild 1 2 3 4 5 Severe)
4. Have you noticed drooping of your eyelids?	No<br>Yes (Mild 1 2 3 4 5 Severe)
5. Have you noticed a change in sensation in your arms or hands?	No<br>Yes (Mild 1 2 3 4 5 Severe)
6. Have you noticed increased pain sensitivity in your hands, arms, shoulder, face or chest wall?	No<br>Yes (Mild 1 2 3 4 5 Severe)
7. Have you noticed a change in temperature sensitivity in your hands, arms and face?	No<br>Yes (Mild 1 2 3 4 5 Severe)
8. Have you noticed a change in power in your arms or hands?	No<br>Yes (Mild 1 2 3 4 5 Severe)

DEPARTMENT OF PHYSIOLOGY, ANATOMY AND GENETICS
Defining Excellence
Oxford Anatomy and Physiology ranked #1 in the QS World University Rankings by subject 2017, 2018

UNIVERSITY OF OXFORD

Symptom Questionnaire

Department of Physiology, Anatomy and Genetics
Sherrington Building
Parks Road
Oxford
OX1 3PT

Radiotherapy side effects	
1. Have you noticed any skin irritation or itchiness in the treatment area?	No<br>Yes (Mild 1 2 3 4 5 Severe)
2. Have you been more tired than usual?	No<br>Yes (Mild 1 2 3 4 5 Severe)
3. Have you had any pain or difficulty swallowing?	No<br>Yes (Mild 1 2 3 4 5 Severe)
4. Have you noticed soreness in your throat?	No<br>Yes (Mild 1 2 3 4 5 Severe)
5. Have you had problems with a hoarse voice?	No<br>Yes (Mild 1 2 3 4 5 Severe)
6. Have you had pain or swelling in your neck?	No<br>Yes (Mild 1 2 3 4 5 Severe)
7. Have you noticed hair loss in the treatment area	No<br>Yes (Mild 1 2 3 4 5 Severe)
8. Have you noticed a new cough	No<br>Yes (Mild 1 2 3 4 5 Severe)
9. Have you experienced any other new symptoms not covered in the questions above?	No<br>Yes - Please give details in free text below:

Overall satisfaction			
1. Are you glad you had this procedure?	Yes	No	Unsure
2. Do you regret having this procedure?	Yes	No	Unsure
3. Would recommend this procedure to someone with your condition?	Yes	No	Unsure

Symptom Questionnaire

Department of Physiology, Anatomy and Genetics
Sherrington Building
Parks Road
Oxford
OX1 3PT
