# Supplementary material for: Protocol of the RADIO-STAR trial: a phase 1 safety and dose finding study of hypofractionated radiotherapy to the stellate ganglia for the treatment of ventricular arrhythmia
Source: BMJ Open. 2026 Feb 25;16(2):e110958. doi: 10.1136/bmjopen-2025-110958 (PMC12959014; doi:10.1136/bmjopen-2025-110958)
Supplement: Supplementary file 5 [file bmjopen-2025-110958.reviewer_comments.pdf]

PEER REVIEW HISTORY

BMJ Open publishes all reviews undertaken for accepted manuscripts. Reviewers are asked to complete a checklist review form and are provided with free text boxes to elaborate on their assessment. These free text comments are reproduced below.

ARTICLE DETAILS

Title (Provisional)

Rational and Design of the RADIO-STAR trial: A Phase 1 safety and dose finding study of Hypofractionated Radiotherapy to the Stellate Ganglia for the treatment of Ventricular Arrhythmia

Authors

Bussmann, Benjamin Mothibe; George, Ben; Robinson, Maxwell; Grist, James; Sukumar, Prabakar; Chinherende, Ebison; Sheerin, Fintan; Enzhil, Veni; Rider, Oliver; Jones, Bleddyn; Sabharwal, Ami; Herring, Neil

---

VERSION 1 - REVIEW

Reviewer **1**

Name **D'Angelo, Lucia**

Affiliation **Yuying Children's Hospital of Wenzhou Medical University, Department of Cardiology**

Date **15-Dec-2025**

COI **None**

This is a well-conceived, clearly articulated, and methodologically robust Phase 1 protocol addressing a significant unmet clinical need in the management of refractory ventricular arrhythmias. The concept of non-invasive stellate ganglion modulation utilising precision radiotherapy is innovative, biologically plausible, and supported by a strong pre-clinical and clinical rationale.

The research question is clearly articulated and appropriate for an early-phase investigation, with a specific focus on safety, feasibility, and dose determination. The methods are sufficiently detailed to facilitate replication, especially regarding radiotherapy planning, dose constraints, and safety monitoring procedures. Ethical considerations, trial registration, transparency concerning funding, and governance arrangements are thoroughly addressed. The authors appropriately avoid overstating efficacy and explicitly recognise the limitations inherent to a Phase 1, single-arm study. The discussion provides a well-considered mechanistic framework while remaining proportionate to the objectives of the study.

The abstract should explicitly indicate the predetermined sample size (n = 13) to ensure comprehensiveness. A succinct statement clarifying that secondary and exploratory outcomes will be analysed descriptively (e.g., within-patient comparisons to baseline) would enhance transparency within the Statistical Methods section. Additional details concerning laboratory assays for circulating catecholamines and neuropeptide Y, such as assay types and sample handling procedures, would facilitate the reproducibility detailed in the Biomarker Methods section. As this pertains to a clinical trial protocol, the inclusion of a SPIRIT checklist, or an explicit confirmation of its utilisation, as supplementary material, would ensure full compliance with the reporting standards of BMJ Open. Minor editorial refinements- such as shortening lengthy sentences and correcting occasional typographical issues- could enhance readability but are not substantively necessary.

Overall, this constitutes a high-quality protocol manuscript that aligns with BMJ Open standards for transparency, ethics, and methodological rigour. With minor revisions as proposed, it is poised to make a valuable contribution to the literature on autonomic neuromodulation and cardiac radiotherapy.

**Reviewer**	**2**
**Name**	**Wertli, Maria M.**
**Affiliation**	**University of Bern, Department of General Internal Medicine**
**Date**	**31-Dec-2025**
**COI**	**None**

BMJopen 2025-110958

Rational and Design of the RADIO-STAR trial: A Phase 1 safety and dose finding study of Hypofractionated Radiotherapy to the Stellate Ganglia for the treatment of Ventricular Arrhythmia

Thank you for the opportunity to review the manuscript of a trial protocol. The RADIO STAR is phase 1 safety and dose finding study. The authors aim to include 13 patients with structural heart disease requiring recurrent implantable cardioverter defibrillator (ICD) therapy for VAs. The intervention is a radiotherapy bilaterally to their stellate ganglia. In this study, the radiotherapy dose will be determined by a pre-specified dose escalation protocol.

Cardiac sympathetic denervation using radiotherapy is an interesting approach to an important clinical issue. The review was conducted following the SPIRIT 2025 checklist for trial protocols and comments are aimed at increasing reproducibility and readability of the study methods.

Abstract:

The primary outcome should be identical to the one published on https://www.isrctn.com/ISRCTN49861434. Please add the treatment-related side effects.

Conclusion: Please restrict the conclusion to the study. Although there is potential, the study's contribution should be toned down.

Methods:

The outcomes should align to the published outcomes on ISRCTN. Please add how treatment related side effects will be assessed and who will be the assessors. How will neurological complications be assessed?

Table 3: Please provide a detailed description on how the outcomes will be assessed (e.g. heart rate variability).

What information will be collected to determine a potential performance bias over 6 months?

Consider adding a schedule of study procedures as given in Appendix 21 A.

Strength and limitations:

Should point out the goal of safety and dose finding.

Discussion

Could the authors expand on the potential safety issues that need to be expected? Given that surgery resulted in up to 50% of patients with neurological effects such as upper limb neuropathic pain, altered sweating, Horner's syndrome and transient hypotension, what are the expected AE based on other studies (e.g., https://pmc.ncbi.nlm.nih.gov/articles/PMC6195178/)

Further, what are the long-term effects of the low dose therapy compared to surgery?

VERSION 1 - AUTHOR RESPONSE

Reviewer: 1
Miss Lucia D'Angelo, Yuying Children's Hospital of Wenzhou Medical University

Comments to the Author:
This is a well-conceived, clearly articulated, and methodologically robust Phase 1 protocol addressing a significant unmet clinical need in the management of refractory ventricular arrhythmias. The concept of non-invasive stellate ganglion modulation utilising precision radiotherapy is innovative, biologically plausible, and supported by a strong pre-clinical and clinical rationale.

The research question is clearly articulated and appropriate for an early-phase investigation, with a specific focus on safety, feasibility, and dose determination. The methods are sufficiently detailed to facilitate replication, especially regarding

radiotherapy planning, dose constraints, and safety monitoring procedures. Ethical considerations, trial registration, transparency concerning funding, and governance arrangements are thoroughly addressed. The authors appropriately avoid overstating efficacy and explicitly recognise the limitations inherent to a Phase 1, single-arm study. The discussion provides a well-considered mechanistic framework while remaining proportionate to the objectives of the study.

The abstract should explicitly indicate the predetermined sample size (n = 13) to ensure comprehensiveness.

Thank you. We have now explicitly stated in the abstract that the predetermined sample size is 13.

A succinct statement clarifying that secondary and exploratory outcomes will be analysed descriptively (e.g., within-patient comparisons to baseline) would enhance transparency within the Statistical Methods section.

We have included a statement on the statistical methods we will use to describe and analyse both primary and secondary endpoints in the under a new ‘statistical analysis’ section

Additional details concerning laboratory assays for circulating catecholamines and neuropeptide Y, such as assay types and sample handling procedures, would facilitate the reproducibility detailed in the Biomarker Methods section.

We agree consistency in sample handling is crucial for consistency in measuring circulating biomarkers. We have thus detailed our sample handling procedures under the ‘Biomarker measurement’ section in the full trial protocol, which will be available as an online supplement. This includes pre-specified use of the NPY assay (EZHNPY-25K, Millipore) along with references of prior use of this assay. Regarding catecholamines, due to their instability in plasma, these are notoriously difficult to quantify accurately. At the time of writing, we are optimising a GCMS (Gas chromatography- Mass Spectrometry) assay which we believe will provide highly specific and sensitive quantification of catecholamines, even at low circulating concentrations. However, as we are still optimising this assay, it was not pre-specified during trial design and is thus not included in the trial protocol.

As this pertains to a clinical trial protocol, the inclusion of a SPIRIT checklist, or an explicit confirmation of its utilisation, as supplementary material, would ensure full compliance with the reporting standards of BMJ Open.

Thank you. We have included a statement regarding SPIRIT guidelines in the ‘methods and analysis’ section and included the checklist as an online supplement

Minor editorial refinements- such as shortening lengthy sentences and correcting occasional typographical issues- could enhance readability but are not substantively necessary.

Thank you. We have proofread again and made appropriate changes to enhance readability.

Overall, this constitutes a high-quality protocol manuscript that aligns with BMJ Open standards for transparency, ethics, and methodological rigour. With minor revisions as proposed, it is poised to make a valuable contribution to the literature on autonomic neuromodulation and cardiac radiotherapy.

Reviewer: 2
Prof. Maria M. Wertli, University of Bern, Cantonal Hospital Baden

Comments to the Author:
Thank you for the opportunity to review the manuscript of a trial protocol. The RADIO STAR is phase 1 safety and dose finding study. The authors aim to include 13 patients with structural heart disease requiring recurrent implantable cardioverter defibrillator (ICD) therapy for VAs. The intervention is a radiotherapy bilaterally to their stellate ganglia. In this study, the radiotherapy dose will be determined by a pre-specified dose escalation protocol.

Cardiac sympathetic denervation using radiotherapy is an interesting approach to an important clinical issue. The review was conducted following the SPIRIT 2025 checklist for trial protocols and comments are aimed at increasing reproducibility and readability of the study methods.

Abstract:
The primary outcome should be identical to the one published on https://www.isrctn.com/ISRCTN49861434. Please add the treatment-related side effects.

Thank you. We have now also added the treatment related side-effects to the abstract.

Conclusion: Please restrict the conclusion to the study. Although there is potential, the study's contribution should be toned down.

Thank you. We have removed the conclusion section in line with the editor's request.

Methods:
The outcomes should align to the published outcomes on ISRCTN. Please add how

treatment related side effects will be assessed and who will be the assessors. How will neurological complications be assessed?

We have clarified how we will assess for treatment related adverse events in table 3. Assessment for all complications, including neurological complications will include patient reported symptoms on questionnaires and detection of clinical examination by trained clinical members of the study team.

Table 3: Please provide a detailed description on how the outcomes will be assessed (e.g. heart rate variability).

We have changed table 3 to expand on how outcomes will be measured.

What information will be collected to determine a potential performance bias over 6 months?

Thank you. We have clarified that as part of study follow up, a record will be kept a of patients' arrhythmia management by their usual clinical team including cardiac ablation procedures, changes in cardiac medications and alterations to ICD programming parameters.

Consider adding a schedule of study procedures as given in Appendix 21 A.

Thank you. The full trial protocol which will be available as an online supplement includes the schedule of events for this study. We have signposted this in the 'Study plan' section.

Strength and limitations:
Should point out the goal of safety and dose finding.

Thank you. We have re-formatted the 'strengths and limitations' section as per the editors request and also included the goals of safety and radiotherapy dose finding.

Discussion
Could the authors expand on the potential safety issues that need to be expected? Given that surgery resulted in up to 50% of patients with neurological effects such as upper limb neuropathic pain, altered sweating, Horner's syndrome and transient hypotension, what are the expected AE based on other studies (e.g., https://pmc.ncbi.nlm.nih.gov/articles/PMC6195178/) Further, what are the long-term effects of the low dose therapy compared to surgery?

Thank you for raising this important consideration. As radiotherapy to the stellate ganglia is completely novel, it is difficult to predict the nature and rate of adverse events. The best available evidence comes form case series of surgical cardiac sympathetic denervation, and we have relied on this to extrapolate potential adverse

effects. Similarly, the nature and frequency of adverse effects related to ionising radiation to the neck are extrapolated from oncological case series. We have elaborated on this in an additional paragraph added to the discussion section.

Reviewer: 1
Competing interests: Not applicable

Reviewer: 2
Competing interests: Not applicable

VERSION 2 - REVIEW

**Reviewer**	**2**
**Name**	**Wertli, Maria M.**
**Affiliation**	**University of Bern, Department of General Internal Medicine**
**Date**	**31-Jan-2026**
**COI**	

I thank the authors for their carefully revised manuscript. I have no additional comments.
